# Supplementary figures and images for: Microtubules oppose cortical actomyosin-driven membrane ingression during C. elegans meiosis I polar body extrusion
Source: PLoS Genet. 2023 Oct 2;19(10):e1010984. doi: 10.1371/journal.pgen.1010984 (PMC10569601; doi:10.1371/journal.pgen.1010984)

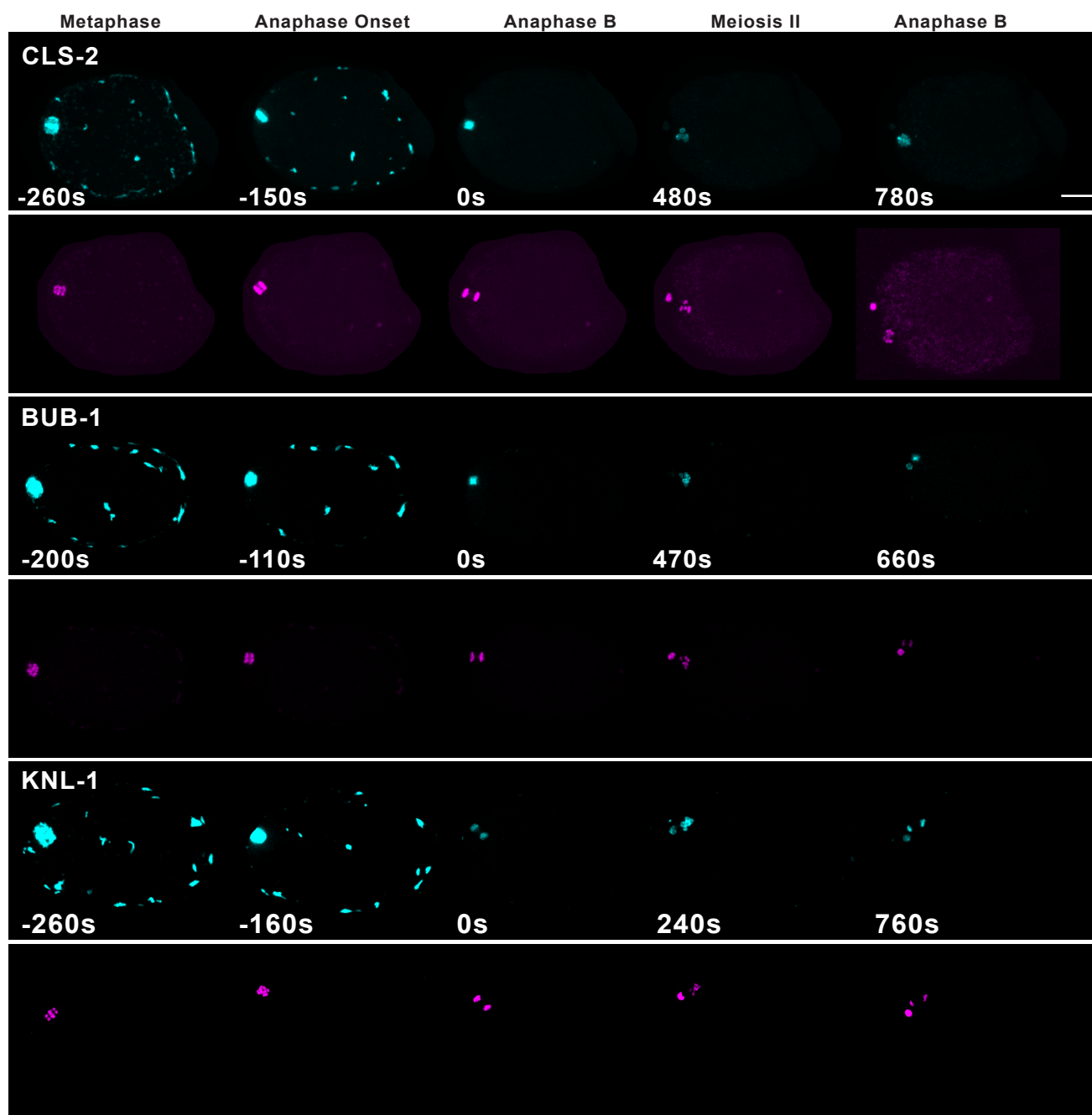

Supplement: S1 Fig — Maximum intensity projections of all focal planes during meiosis I in live ex utero oocytes expressing mCherry::H2B (magenta) to mark chromosomes (lower rows) and GFP (cyan) fusions (upper rows) to CLS-2 (A), BUB-1 (B), and KNL-1 (C). All three proteins were associated with the oocyte spindle and chromosomes, and were present in sub-cortical patches, during meiosis I, but were associated only with the spindle and chromosomes, and not present in sub-cortical patches, during meiosis II. (PDF) [file pgen.1010984.s001.pdf]

A.

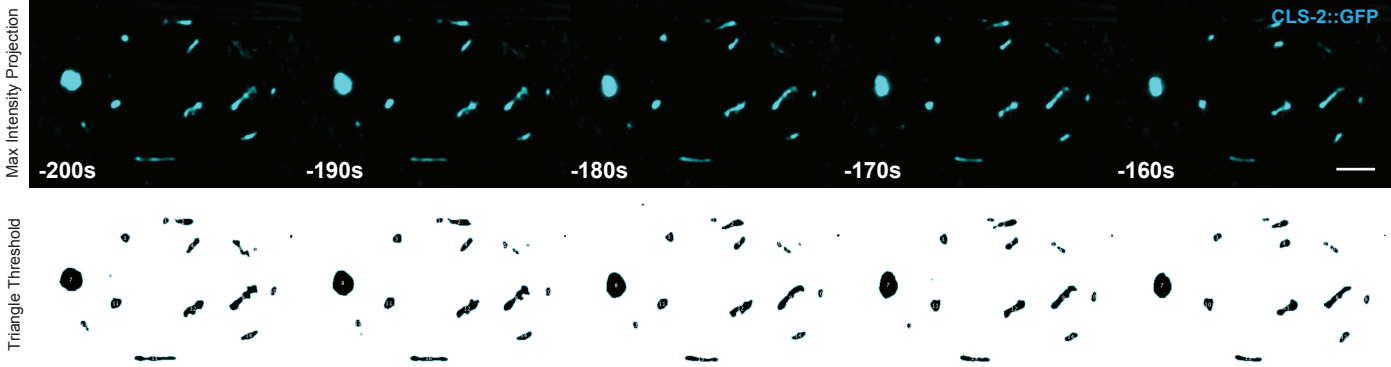

B.

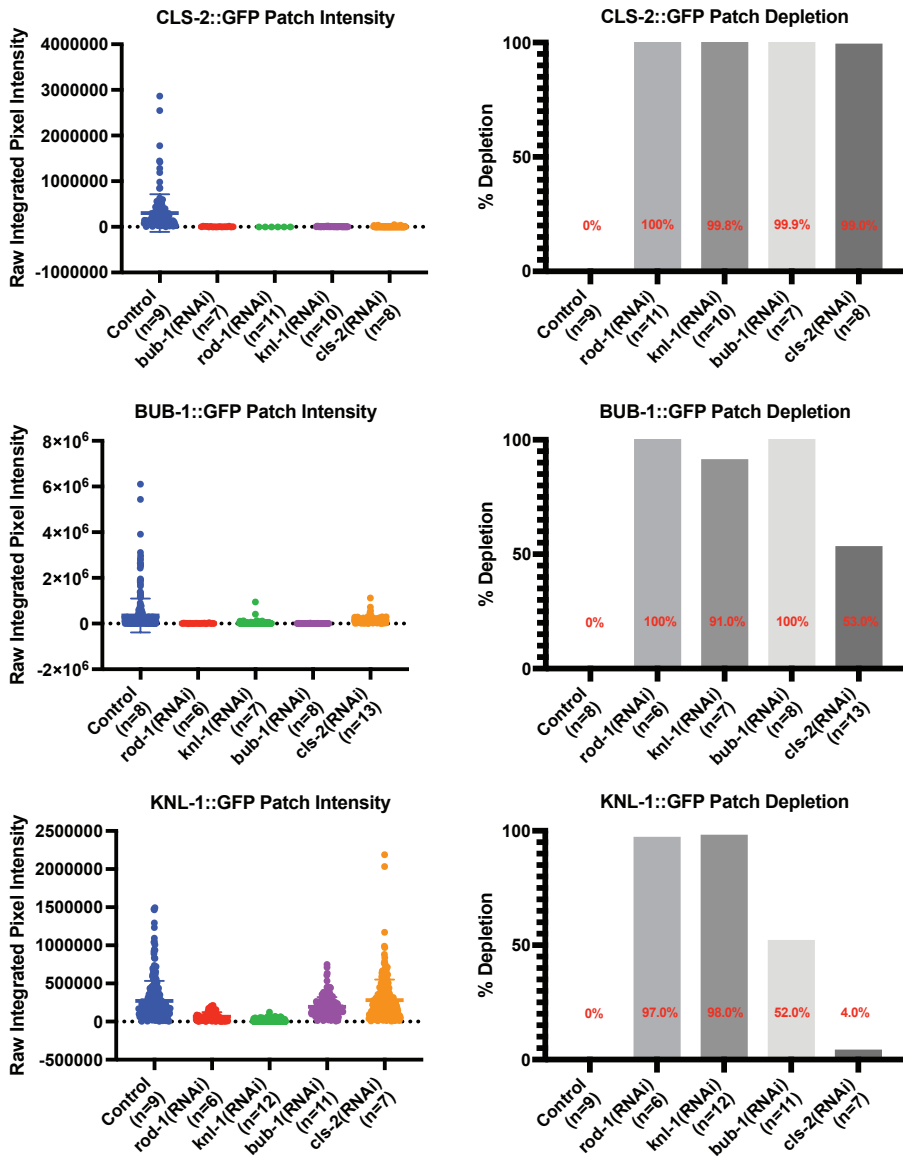

Supplement: S2 Fig — (A) Maximum intensity projections (MIPs) of the five surface-most focal planes (upper row), and the same MIPs after a Triangle threshold filter to identify sub-cortical patches (lower row) in an ex utero oocyte expressing a CLS-2::GFP fusion, over 5 time points preceding the start of anaphase B. (B) Quantification of threshold-limited sub-cortical patch intensities, after manually removing spindle associated signal, in control and mutant ex utero oocytes expressing GFP fusions to CLS-2, BUB-1 and KNL-1 as indicated, showing scatter plots for raw integrated pixel intensity and bar graphs for per cent depletions. See S1 File for raw data on patch quantification. (PDF) [file pgen.1010984.s002.pdf]

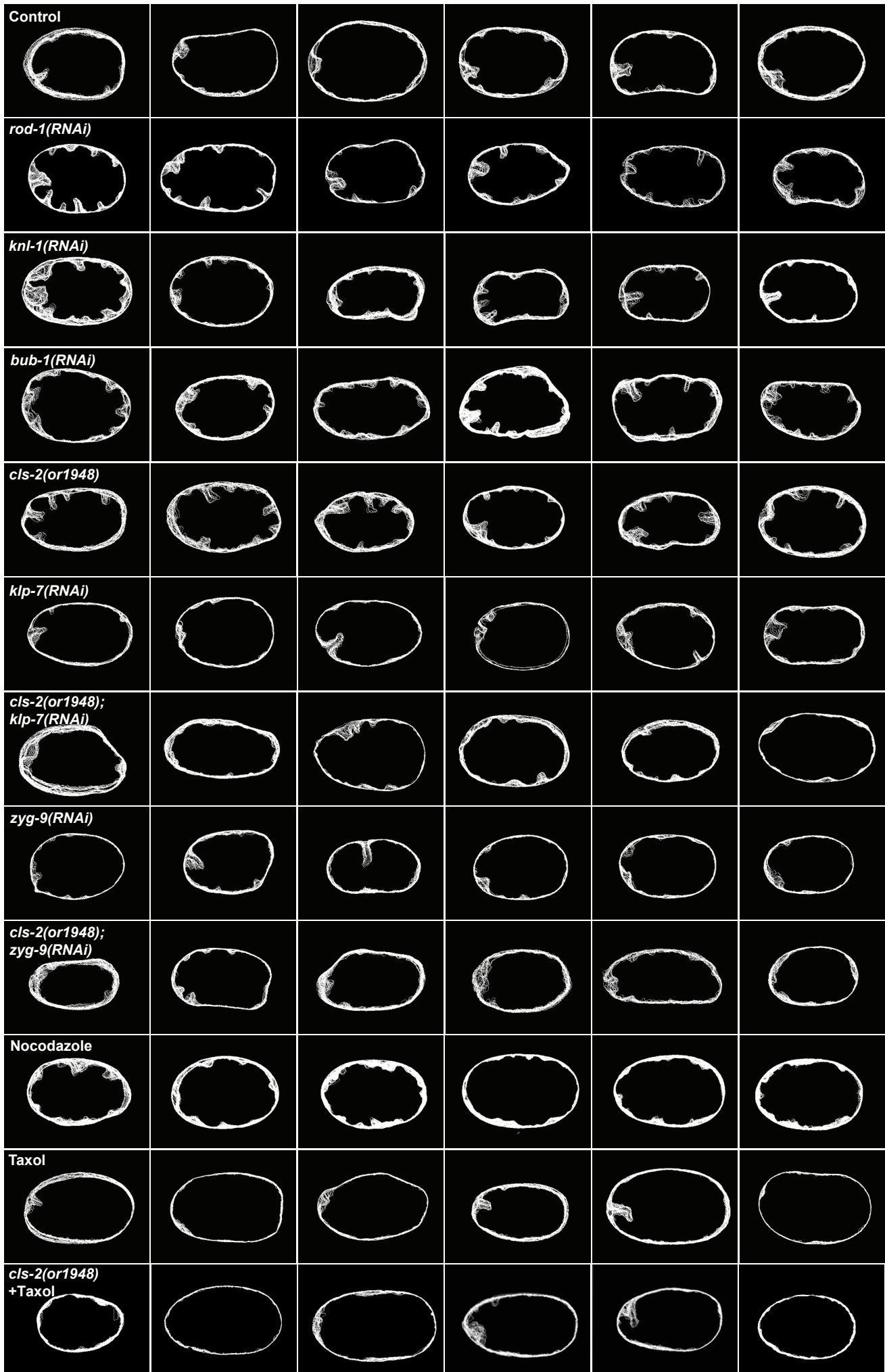

Supplement: S3 Fig — Time projections of single central focal planes throughout meiosis I anaphase B from six ex utero oocytes of each genotype or condition, all expressing GFP::PH (cyan) and mCherry::H2B (not shown) to mark oocyte membranes and chromosomes, respectively. (PDF) [file pgen.1010984.s003.pdf]

A.

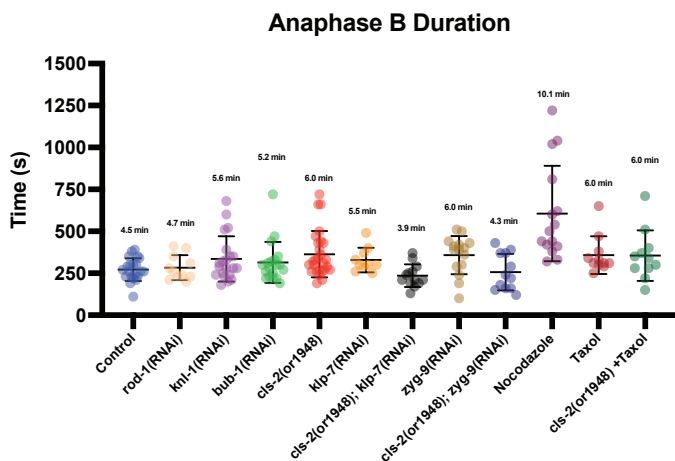

B.

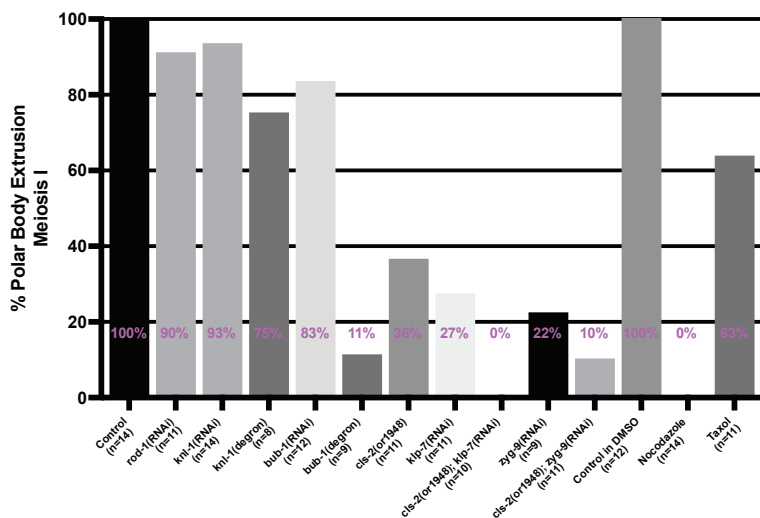

C. *C. elegans* oocyte model of a convex hull and contour to measure furrow length

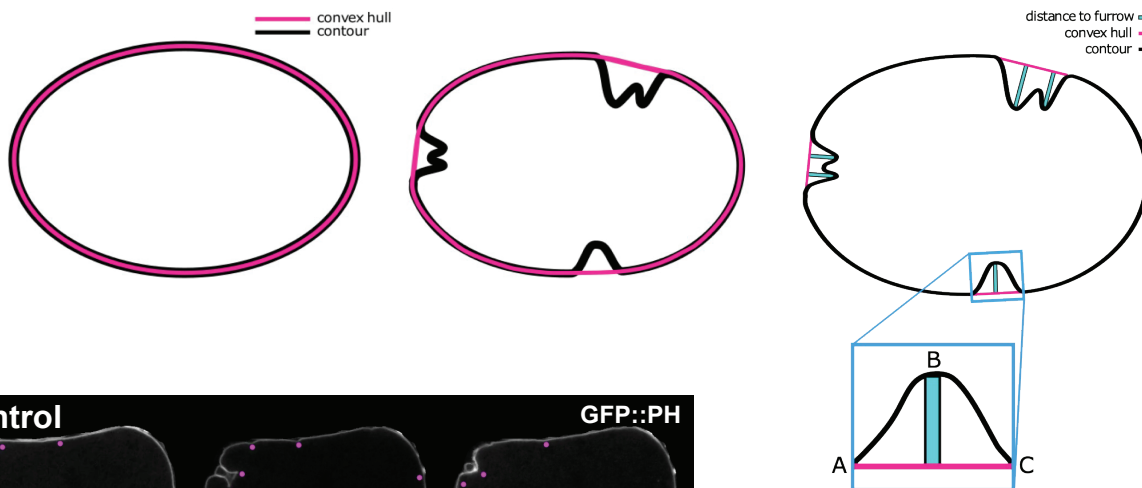

D.

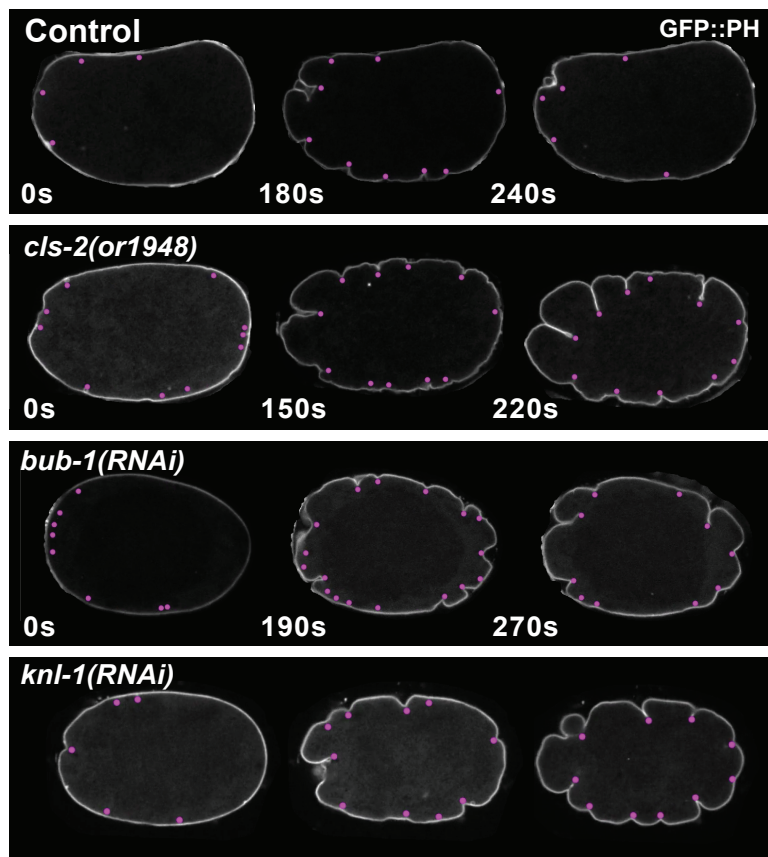

E.

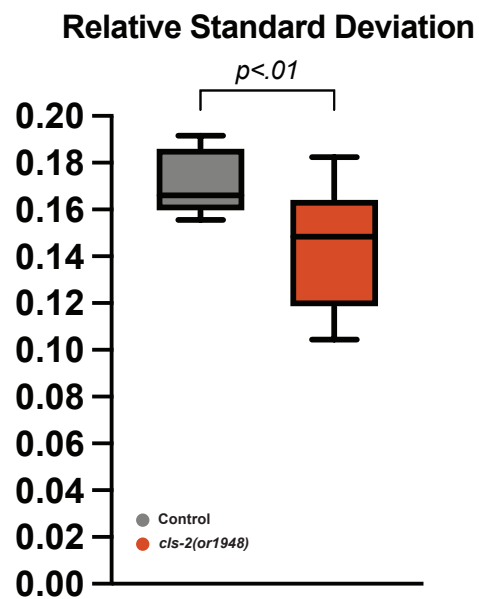

Supplement: S4 Fig — (A) Anaphase B duration in oocytes of each genotype or condition, with mean and standard deviation indicated. Mean in minutes are shown above each scatter plot. See S5 File for raw data. (B) Bar graphs indicating number of embryos scored and percent of each genotype or condition in which polar body extrusion after meiosis I was successful, with some mCherry::H2B signal detected in an intact polar outside of the oocyte after the completion of meiosis I. See S5 File for raw data (C) Schematic of a convex hull and contour model to measure furrow lengths in C. elegans oocytes. Defects in the contour are shown in middle oocyte and are quantified using a distance formula (see Materials and Methods). (D) Single focal planes of live control and mutant oocytes strains that express GFP::PH (white) and mCherry::H2B (not shown) processed for scoring ingression. Pink dots indicate furrows that are computationally counted and measured in length (see Materials and Methods). (E) Relative standard deviation of five surface-most focal planes ten seconds after start of anaphase B in ex utero control and cls-2 mutant oocytes. Maximum intensity projections images of surface-most five focal planes from ex utero oocytes 10 seconds after anaphase B onset were generated, excluding a circular region with an 80 (11.75 μm) pixel radius surround the spindle were hand segmented and subjected for quantification of mean pixel intensity and pixel intensity standard deviation. Relative standard deviation was calculated by dividing standard deviation by the mean. See S4 File for raw data. (PDF) [file pgen.1010984.s004.pdf]

A.

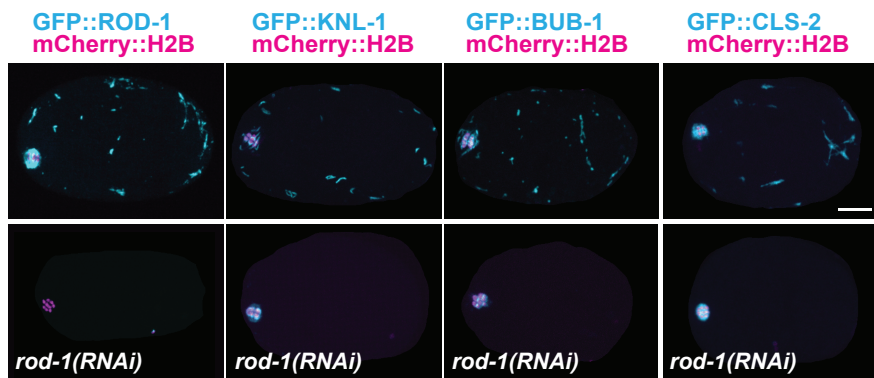

D.

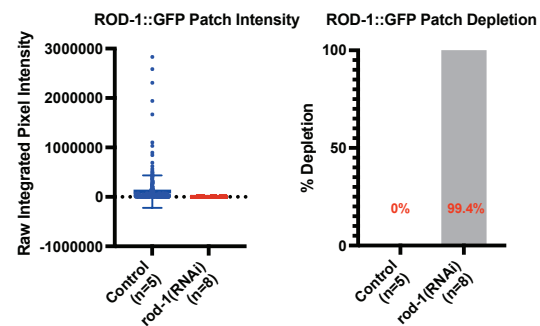

B. Control

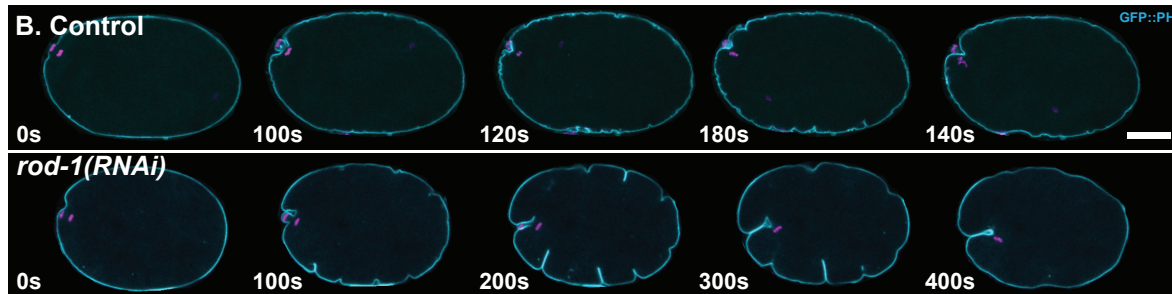

C. Anaphase B Ingressions Over Normalized Time in *rod-1(RNAi)*

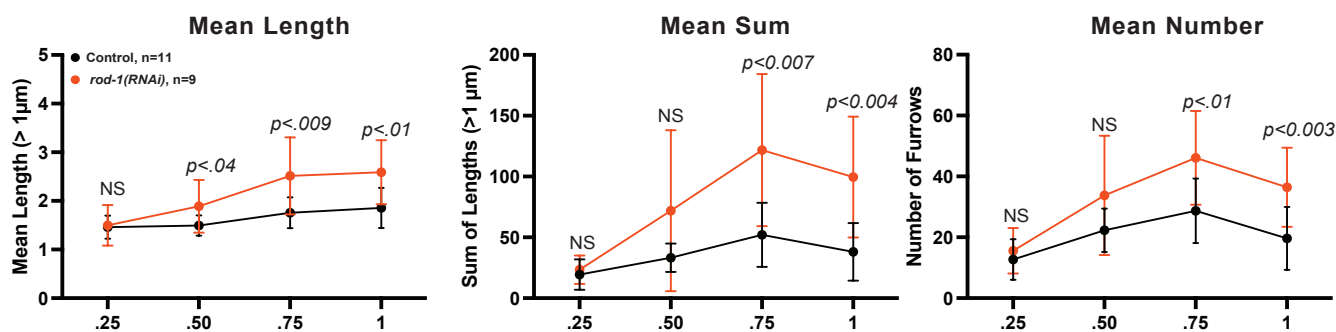

Supplement: S5 Fig — (A) Maximum intensity projections (MIPs) of the five surface-most focal planes, merged with 5 internal focal planes that include most of the chromosomes, in live control (upper row) or rod-1(RNAi) (lower row) ex utero oocytes expressing mCherry::H2B to mark chromosomes (magenta) and a GFP fusion to ROD-1, CLS-2, BUB-1 or KNL-1 (cyan). (B) Selected and merged focal planes from ex utero oocytes during meiosis I anaphase B that express GFP::PH (cyan) and mCherry::H2B (magenta) to mark the plasma membrane and chromosomes, respectively. A single central focal plane is shown for the membrane, merged with a maximum intensity projection of 5 consecutive internal focal planes that encompass most of the oocyte chromosomes. (C) Quantification of the mean length of membrane ingressions that were 1μm or more in length, the mean sum of lengths, and the mean number of membrane ingressions, over normalized anaphase B time in control and rod-1(RNAi) oocytes. The mean length and mean sum of lengths, but not the number, of ingressions were significantly increased in rod-1 mutant oocytes. (D) Quantification of threshold-limited sub-cortical patch intensities in control and rod-1(RNAi) oocytes expressing GFP fused to ROD-1 and mCherry::H2B, after manually removing spindle associated signal, showing scatter plots for raw integrated pixel intensities and bar graphs for per cent depletion. (PDF) [file pgen.1010984.s005.pdf]

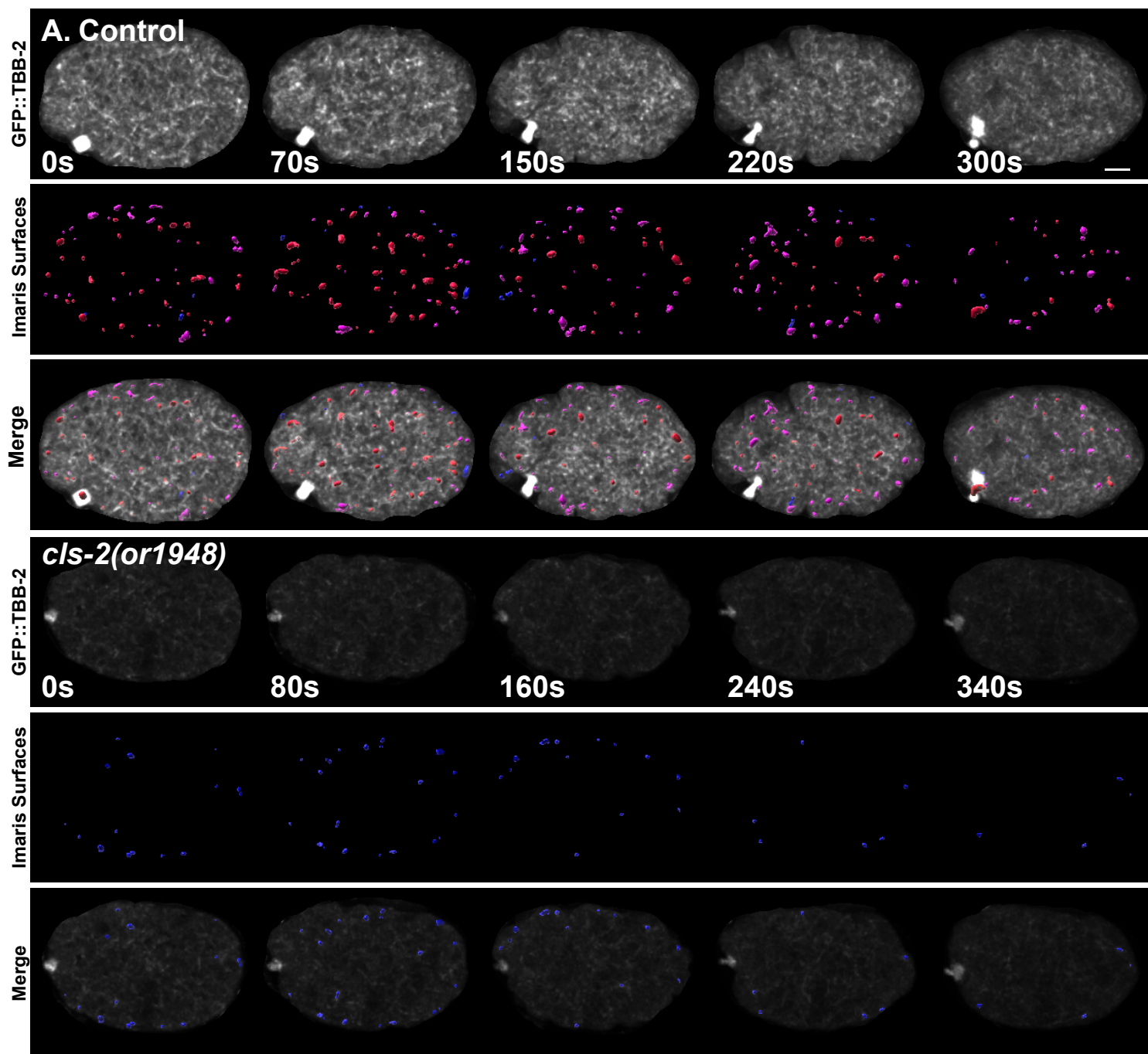

**B.**

**Control oocyte**

***cls-2(or1948)* oocyte**

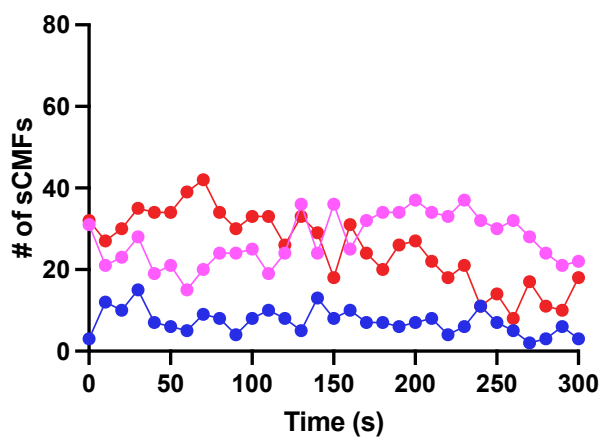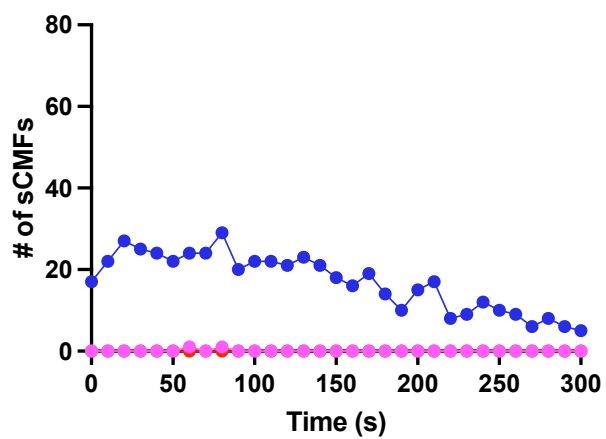

Supplement: S6 Fig — (A) Merged maximum intensity projections of ex utero control and cls-2(or1948) oocytes that express GFP::TBB-2 (white) to mark microtubules, and mCherry::H2B to mark chromosomes (not shown). Top rows for control and cls-2(or1948) oocytes show GFP::TBB-2 (white); middle rows show color-coded Imaris surfaces, based on our cutoffs for weak (blue), medium (purple), and strong (red) sCMFs (see Materials and Methods); bottom rows show merges of GFP::TBB-2 and Imaris surfaces. (B) Quantification of weak, medium and strong sCMFs in single control and cls-2(or1948) oocytes during anaphase B. The cls-2(or1948) oocyte shown has mostly weak sCMFs, with a loss of medium and strong sCMFs throughout anaphase B. (PDF) [file pgen.1010984.s006.pdf]

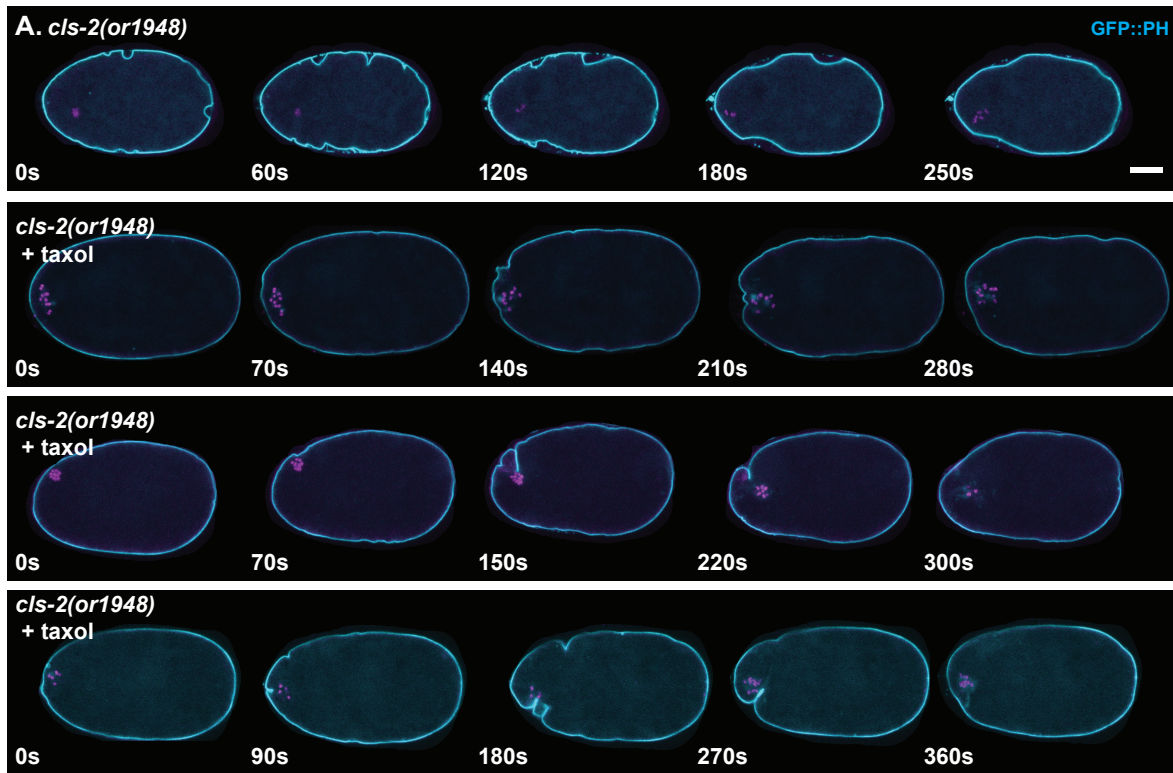

**B. Anaphase B Ingressions Over Normalized Time in Taxol Treated Oocytes**

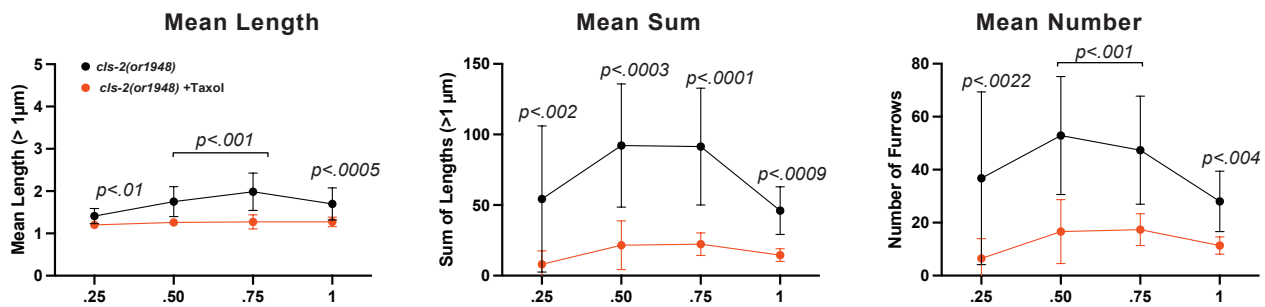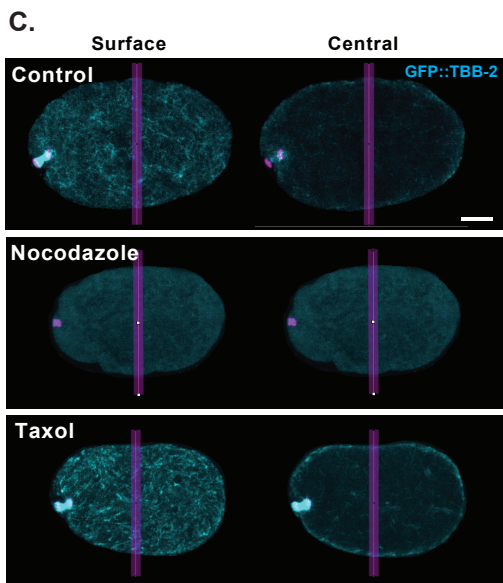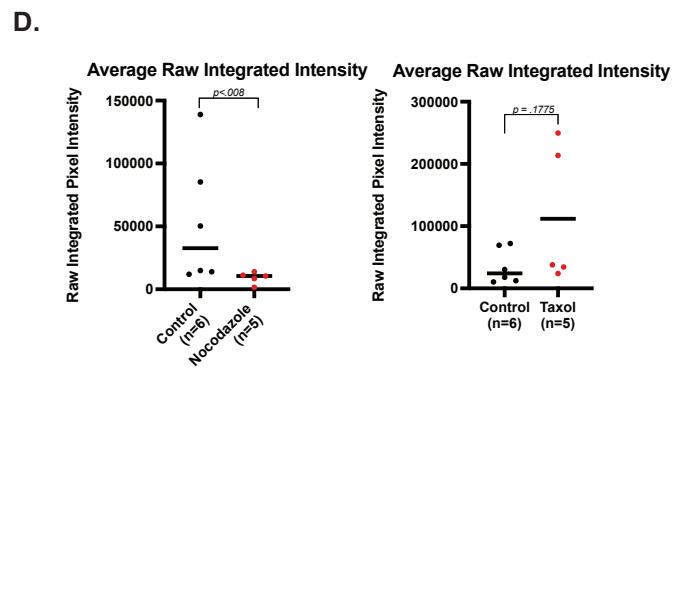

Supplement: S7 Fig — (A) Selected and merged focal planes from live control (DMSO treated; upper row) and taxol-treated (three lower rows) ex utero cls-2(or1948) oocytes, during meiosis I anaphase B, that express GFP::PH (cyan) and mCherry::H2B (magenta) to mark the plasma membrane and chromosomes, respectively. A single central focal plane is shown for the membrane, merged with a maximum intensity projection of 5 consecutive internal focal planes that encompass most of the oocyte chromosomes. (B) Quantification of the mean length of membrane ingressions that were 1μm or more in length, the mean sum of lengths, and the mean number of membrane ingressions, over normalized anaphase B time in control and taxol-treated oocytes. The mean length, mean sum of lengths, and mean number of ingressions were all significantly decreased in taxol-treated cls-2(or1948) oocytes. (C) Five surface-most focal planes (left column) or five central focal planes (middle column), merged with five internal focal planes that include most of the chromosomes, from live ex utero control, nocodazole-treated and taxol-treated oocytes expressing GFP::TBB-2 and mCherry::H2B to mark microtubules and chromosomes, at ¾ of normalized time through anaphase B. Surface (black) and central (red) line scans (right column) are from vertical magenta lines in oocyte images. Scale bar = 10 μm. (D) Quantification of microtubule levels in control, nocodazole-treated and taxol-treated oocytes. See S1 File for raw data on microtubule levels comparisons. (PDF) [file pgen.1010984.s007.pdf]

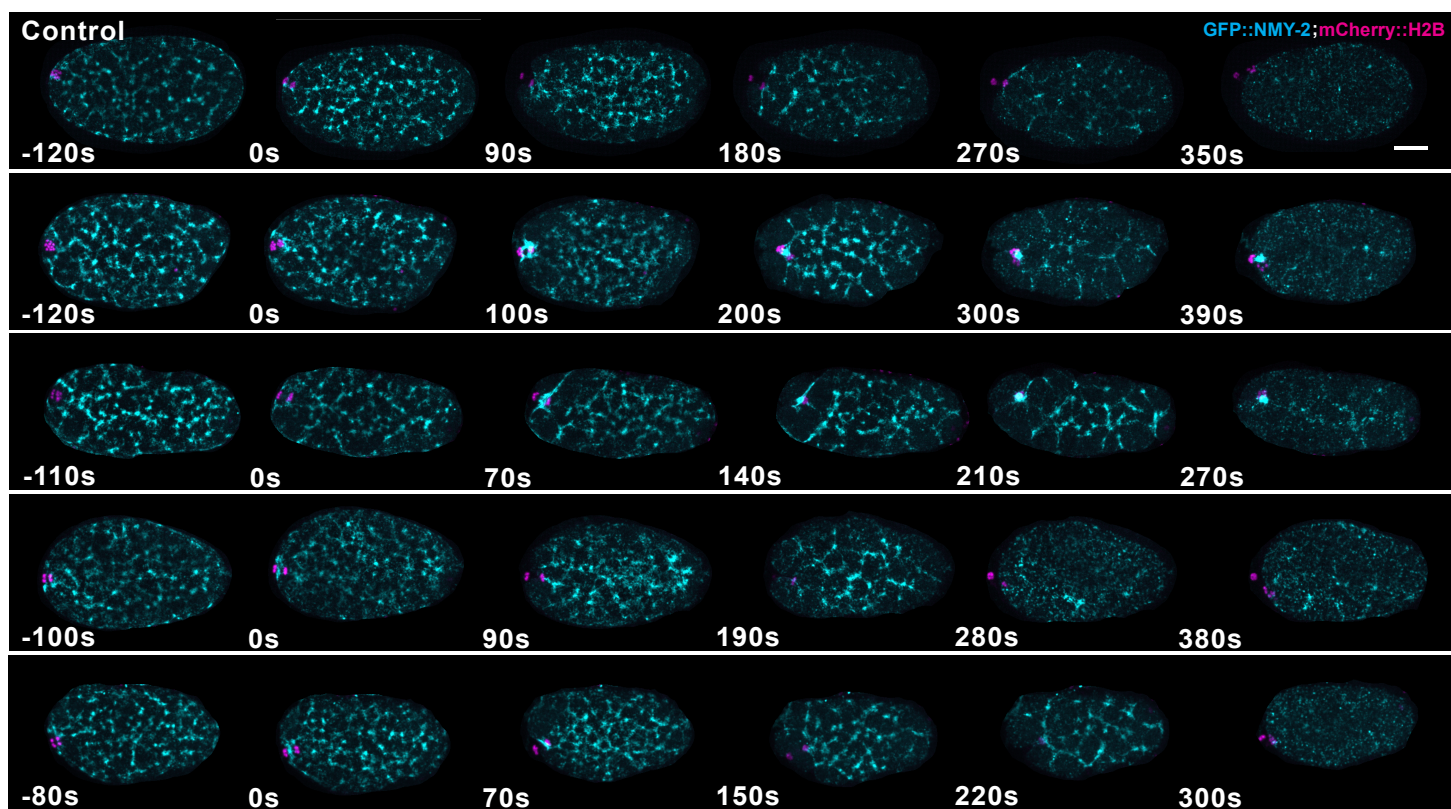

Supplement: S8 Fig — Merged maximum intensity projections (MIPs) during meiosis I anaphase B of five ex utero control oocytes expressing NMY-2::GFP (cyan) and mCherry::H2B (magenta) to mark NMY-2/non-muscle myosin II and chromosomes, respectively. MIPs of five surface-most focal planes showing non-muscle myosin are merged with MIPs of five consecutive internal focal planes that encompass most of the oocyte chromosomes, visible at the left, anterior end of each oocyte. A roughly even but variable and dynamic network of cortical NMY-2::GFP foci persists throughout most of anaphase B until dissipating near the end. (PDF) [file pgen.1010984.s008.pdf]

*cls-2(or1948)*

GFP::NMY-2;mCherry::H2B

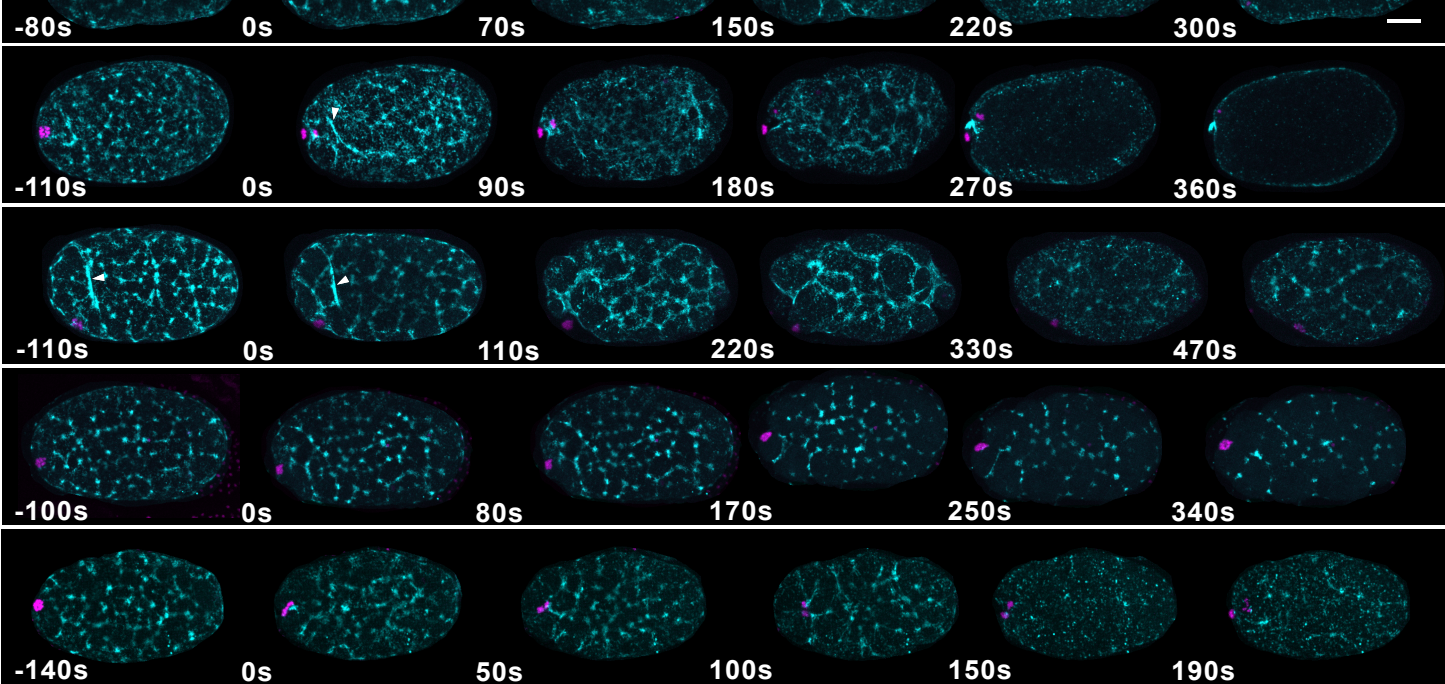

Supplement: S9 Fig — Merged maximum intensity projections (MIPs) during meiosis I anaphase B of five ex utero cls-2(or1948) oocytes expressing NMY-2::GFP (cyan) and mCherry::H2B (magenta) to mark NMY-2/non-muscle myosin II and chromosomes, respectively. MIPs of five surface-most focal planes showing non-muscle myosin are merged with MIPs of five consecutive internal focal planes that encompass most of the oocyte chromosomes, visible at the left, anterior end of each oocyte. Abnormal linear arrays of NMY-2::GFP foci are present in some cls-2 mutant oocytes (arrowheads). (PDF) [file pgen.1010984.s009.pdf]

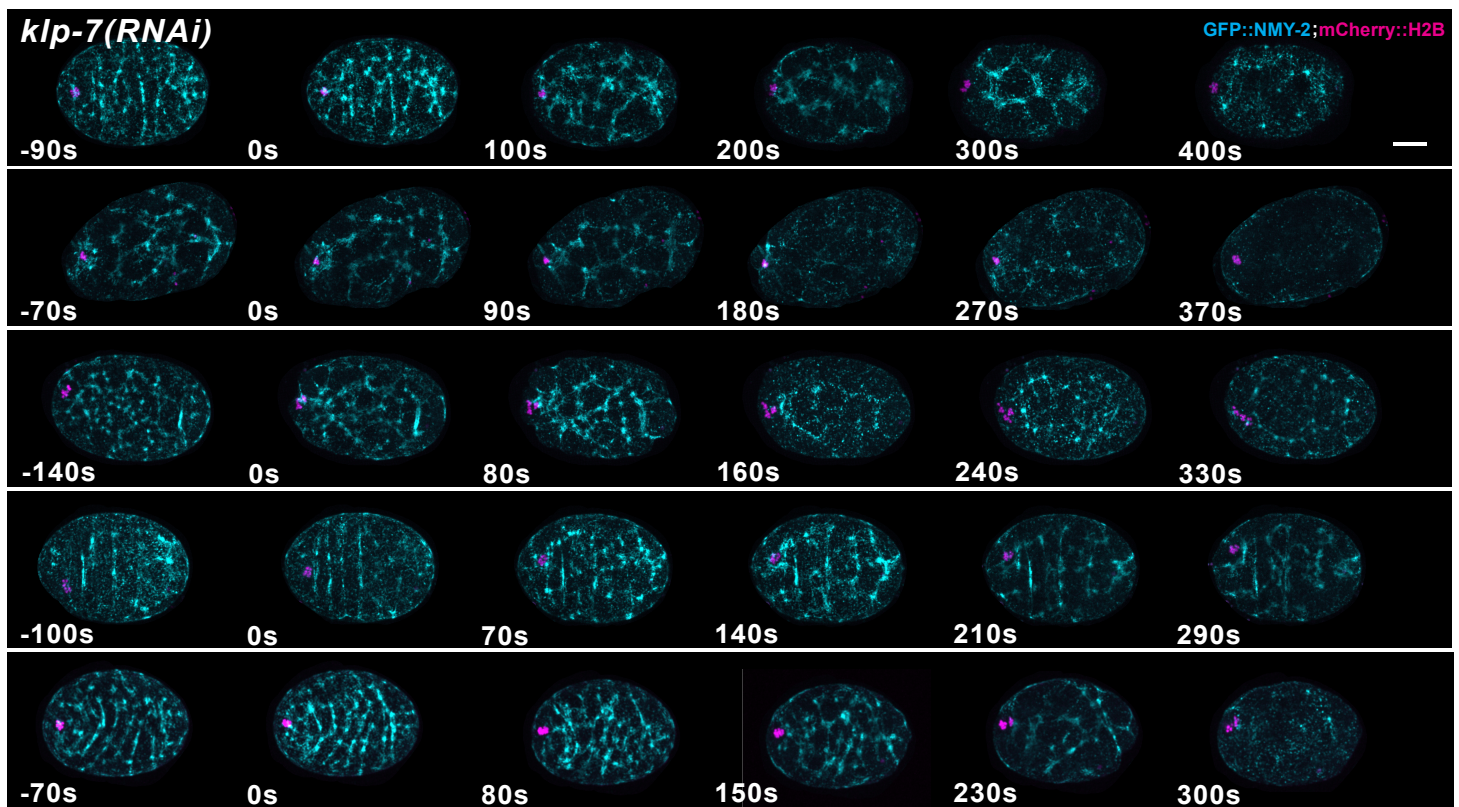

Supplement: S10 Fig — Merged maximum intensity projections (MIPs) during meiosis I anaphase B of five ex utero klp-7(RNAi) oocytes expressing NMY-2::GFP (cyan) and mCherry::H2B (magenta) to mark NMY-2/non-muscle myosin II and chromosomes, respectively. MIPs of five surface-most focal planes showing non-muscle myosin are merged with MIPs of five consecutive focal planes that encompass most of the oocyte chromosomes,visible at the left, anterior end of each oocyte. (PDF) [file pgen.1010984.s010.pdf]

***zyg-9(RNAi)***

GFP::NMY-2;mCherry::H2B

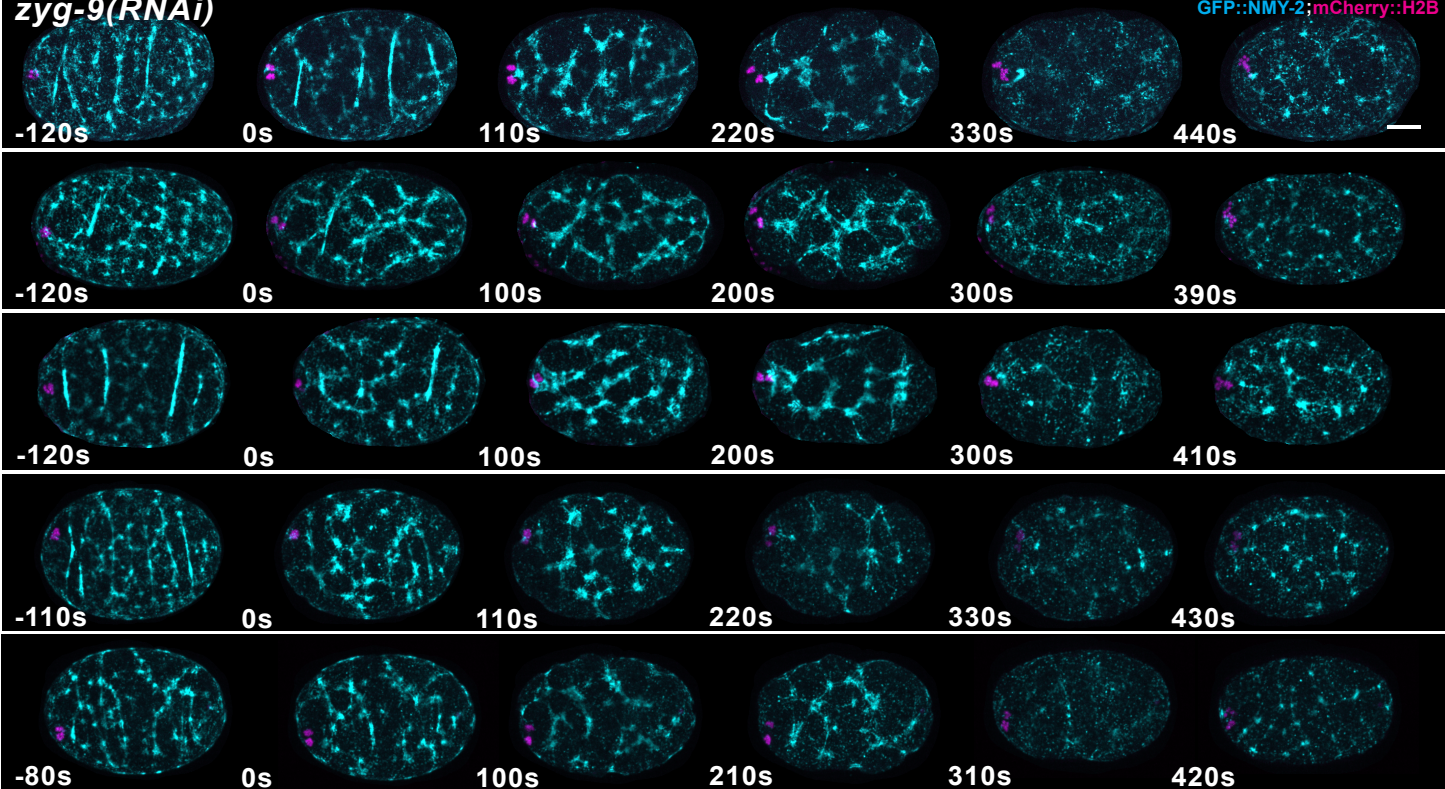

Supplement: S11 Fig — Merged maximum intensity projections (MIPs) during meiosis I anaphase B of five ex utero zyg-9(RNAi) oocytes expressing NMY-2::GFP (cyan) and mCherry::H2B (magenta) to mark NMY-2/non-muscle myosin II and chromosomes, respectively. MIPs of five surface-most focal planes showing non-muscle myosin are merged with MIPs of five consecutive internal focal planes that encompass most of the oocyte chromosomes, which are visible at the left, anterior end of each oocyte. (PDF) [file pgen.1010984.s011.pdf]

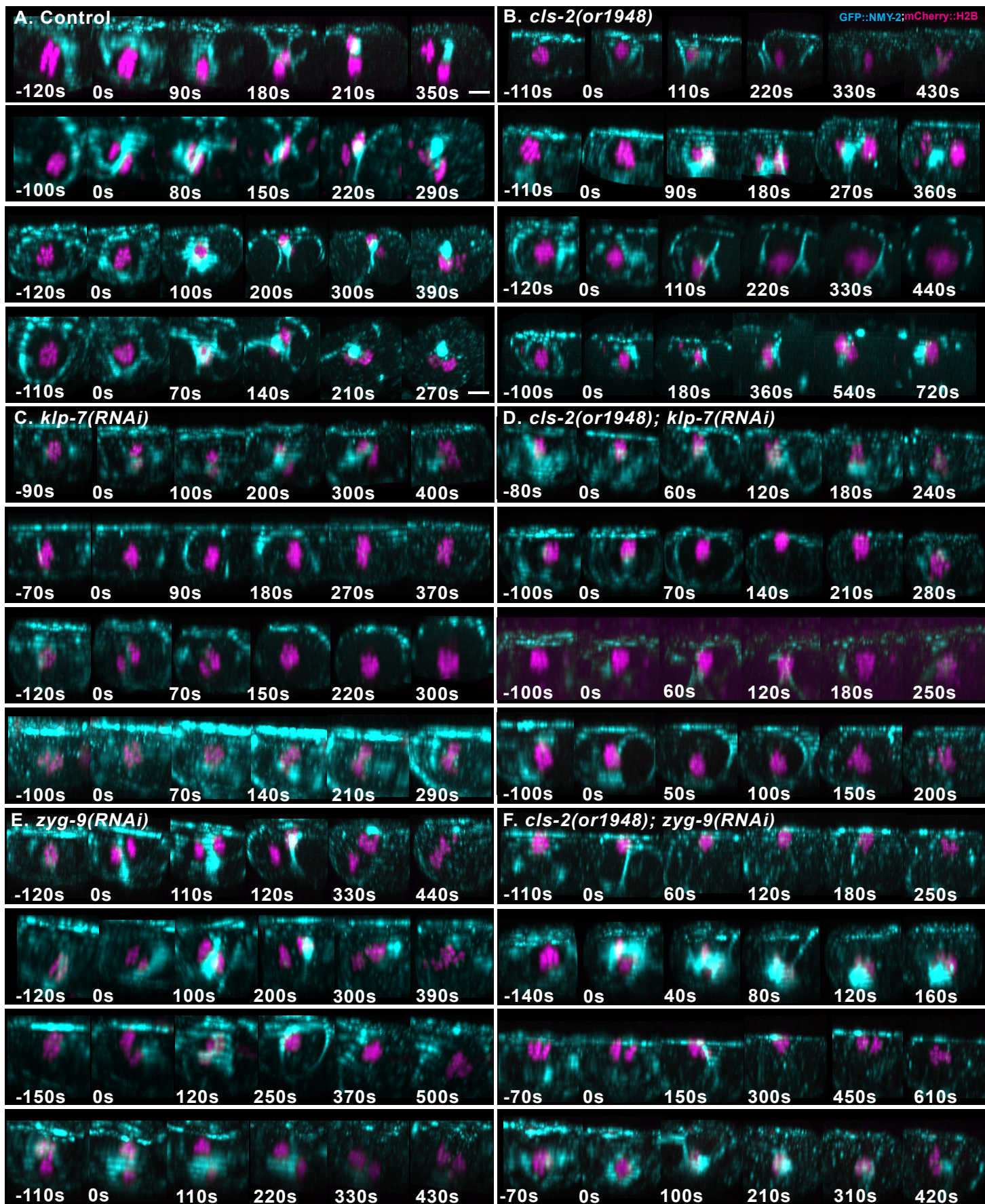

Supplement: S12 Fig — Projections of all focal planes after Imaris-mediated rotation to obtain ring-centric, more oocyte-end-on views of contractile ring dynamics in four each of control and mutant oocytes expressing GFP::NMY-2 (cyan) and mCherry:H2B (magenta) to mark NMY-2/non-muscle myosin II and chromosomes, respectively, in control and mutant oocytes (see Materials and Methods). (PDF) [file pgen.1010984.s012.pdf]

Control Oocyte 1

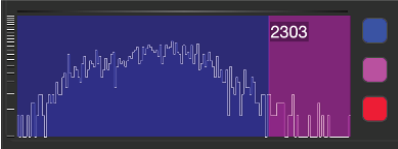

Control Oocyte 2

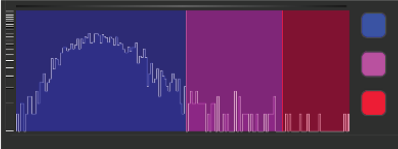

Control Oocyte 3

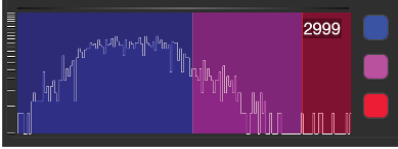

Supplement: S13 Fig — Histograms are based on the mean intensity of all surfaces. Each surface represents a single sCMF. The histograms are automatically made in Imaris throughout the course of image processing. The classification threshold between class for weak and medium 2303 was set to separate the left most mountain, best represented in Control Oocyte 1 and Control Oocyte 2. Control oocyte 1 was a darker example of the three presented, and it shows a high frequency of weak sCMFs. Control oocyte 2 was a brighter example, and the thresholding cutoffs are represented with the thresholding covering the whole mountain within the weak sCMF range, and lower frequency for medium, and strong sCMFs. The weak class does not cover the whole left-most mountain in control oocyte 3, a very bright example, and sCMFs with high intensity in the mountain are classified as medium. But the bright foci outside the mountain are classified as the strong class with the threshold 2999. (PDF) [file pgen.1010984.s013.pdf]

A.

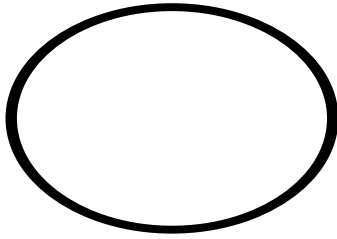

B.

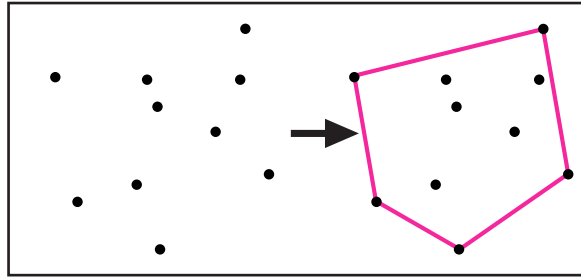

C.

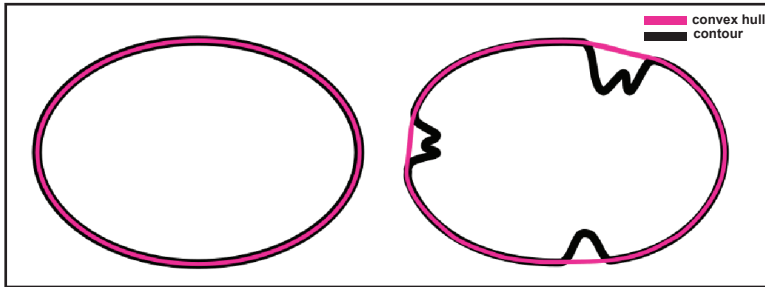

D.

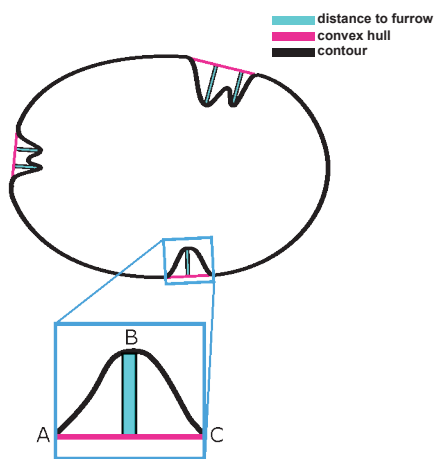

E.

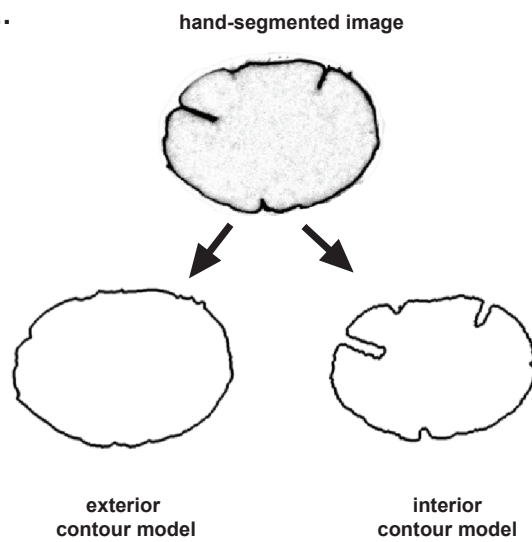

Supplement: S14 Fig — (A) Contour model of an oocyte cortex without ingressions. (B) Visual description of a convex hull. (C) Contour and convex hull model of the oocyte cortex without (left) and with (right) ingressions. (D) Schematic for the calculation of ingression lengths from the line joined by intersection points A and C to any point B along the contour. (E) Inverting the intensity-based segmentation of the cortex allows for a better contour model of the ingrressions. The contour on the bottom left uses the intensity-based threshold of the cortex fluorescence as the input for the contour model (exterior contour model), but poorly captures the narrow ingresions. Instead we used the cortex intensity as a border and the inverted interior as the input for the contour model (bottom right). (PDF) [file pgen.1010984.s014.pdf]
