# Supplementary material for: Microtubules oppose cortical actomyosin-driven membrane ingression during C. elegans meiosis I polar body extrusion
Source: PLoS Genet. 2023 Oct 2;19(10):e1010984. doi: 10.1371/journal.pgen.1010984 (PMC10569601; doi:10.1371/journal.pgen.1010984)
Supplement: S1 Table — (PDF) [file pgen.1010984.s023.pdf]

EU3228	cpIs53 [mex-5p::GFP-C1::PLC(delta)-PH::tbb-2 3'UTR + unc-119 (+)] II; itIs37[pie1p::mCherry::H2B::pie-1 3'UTR + unc-119(+)] IV
EU3229	cpIs53 [mex-5p::GFP-C1::PLC(delta)-PH::tbb-2 3'UTR + unc-119 (+)] II; cls-2(or1948) /qC1[qIs26] III; itIs37[pie1p::mCherry::H2B::pie-1 3'UTR + unc-119(+)] IV
EU2942	ruIs57[pie-1p::GFP::tubulin + unc-119(+)]; itIs37[pie-1p::mCherry::H2B::pie-1 3'UTR + unc-119(+)] IV
EU3025	cls-2(or1948) /qC1[qIs26] III ; ruIs57[pie-1p::GFP::tubulin + unc-119(+)]; itIs37[pie-1p::mCherry::H2B::pie-1 3'UTR + unc-119(+)] IV
EU3030	ijmSi3 [pJD342/pJD330; ChrI_5'mex-5_cls-2reenc::GFP_3'tbb-2; cb-unc-119(+)] I; unc-119(ed3) III?; itIs37[pie-1p::mCherry::H2B::pie-1 3'UTR + unc-119(+)] IV
EU3382	ddIs68 [bub-1::TY1::EGFP::3xFLAG(92C12) + unc-119(+)]; itIs[pie-1p::mCherry::H2B::pie-1 3'UTR + unc-119(+)] IV;
OD3075	*knl-1(lt53[knl-1::GFP::tev::loxP::3xFlag])) III; ltIs37 [pAA64; pie-1p::mCh::his-58 + unc-119(+)] IV*
EU3079	cls-2(or1948) /qC1[qIs26] III; itIs37[pie-1p::mCherry::H2B::pie-1 3'UTR + unc-119(+)] IV; nmy-2(cp13[nmy-2::gfp + LoxP]) I
EU3080	itIs37[pie-1p::mCherry::H2B::pie-1 3'UTR + unc-119(+)] IV; nmy-2(cp13[nmy-2::gfp + LoxP]) I
EU3422	ddIs68 [bub-1::TY1::EGFP::3xFLAG(92C12) + unc-119(+)] I; ltSi1[pOD809/pJE110; Pknl-1::KNL-1reencoded::RFP; cb-unc-119(+)]II; unc-119(ed3) III
EU3423	ijmSi3 [pJD342/pJD330; ChrI_5'mex-5_cls-2reenc::GFP_3'tbb-2; cb-unc-119(+)] I; unc-119(ed3) III?; ltSi264[pOD/pTK011; Pbub-1::BUB-1 reencoded::RFP; cb-unc-119(+)]II; unc-119(ed3)III
EU3424	ijmSi3 [pJD342/pJD330; ChrI_5'mex-5_cls-2reenc::GFP_3'tbb-2; cb-unc-119(+)] I; unc-119(ed3) III? ; ltSi1[pOD809/pJE110; Pknl-1::KNL-1reencoded::RFP; cb-unc-119(+)]II; unc-119(ed3) III
GCP529	*rod-1(lt62[gfp::rod-1]) IV; ltIs122[pAA64; Ppie-1::mCherry::his-58; cb-unc-119(+)]; unc-119(ed3) III*
EU3440	bub-1[or1960(bub-1::degron)] I; knl-1(lt53[knl-1::GFP::tev::loxP::3xFlag]) III; ltIs37[pie-1p::mCherry::H2B, unc-119(+)]; ieSi38[sun-1p::TIR1::mRuby::sun-1 3' UTR + Cbr-unc-119(+)] IV. (may contain unc-119(ed3) III
EU3442	knl-1(or1997[knl-1::degron]) III; ddIs68 [bub-1::TY1::EGFP::3xFLAG(92C12) + unc-119(+)]. itIs37[pie-1p::mCherry::H2B, unc-119(+)]; ieSi38[sun-1p::TIR1::mRuby::sun-1 3' UTR + Cbr-unc-119(+)] IV. (may contain unc-119(ed3) III)
EU3444	knl-1(lt53[knl-1::GFP::tev::loxP::3xFlag]), cls-2(or1998[cls-2::degron]) III; ieSi38[sun-1p::TIR1::mRuby::sun-1 3' UTR + Cbr-unc-119(+)] IV. (may contain unc-119(ed3) III)
EU3445	cls-2(or1999[cls-2::degron]) III; ddIs68 [bub-1::TY1::EGFP::3xFLAG(92C12) + unc-119(+)]. itIs37[pie-1p::mCherry::H2B, unc-119(+)]; ieSi38[sun-1p::TIR1::mRuby::sun-1 3' UTR + Cbr-unc-119(+)] IV. (may contain unc-119(ed3) III)
