# Supplementary material for: Microtubules oppose cortical actomyosin-driven membrane ingression during C. elegans meiosis I polar body extrusion
Source: PLoS Genet. 2023 Oct 2;19(10):e1010984. doi: 10.1371/journal.pgen.1010984 (PMC10569601; doi:10.1371/journal.pgen.1010984)
Supplement: S6 File — (DOCX) [file pgen.1010984.s029.docx]

**Phylogenetic diversity in oocyte meiotic cell division mechanisms**

Oocyte meiotic cell division has been investigated most extensively in mammals, especially mouse and more recently human, but also in *C. elegans*, *Drosophila* and *Xenopus*. All are alike in that oocyte meiotic spindle assembly is acentrosomal. Moreover, as in vertebrates, *C. elegans* oocyte chromosomes move toward the cortex as the spindle assembles, and the spindle ultimately becomes bipolar and oriented orthogonally to the cell cortex. However, *C. elegans* and vertebrate oocytes differ in many ways, including the roles played by microfilaments and microtubules during spindle assembly and movement, and during polar body extrusion (1).

In vertebrate oocytes, meiotic spindle assembly and positioning, and more recently even chromosome capture by microtubules, all require or depend extensively on actin microfilaments (2,3). In mouse oocytes*,* the acentrosomal spindles assemble centrally and then move along the bipolar spindle axis until the leading pole reaches the cortex. Spindle movement to the cortex requires a branched network of cytoplasmic actin, and a non-muscle myosin that becomes enriched at the leading pole, which upon arrival becomes tethered to the cortex. Subsequently the oocyte chromosomes, independently of microtubules and through the small GTPase Ran, can induce the formation both of an actin cap overlying the proximal pole, and of a non-muscle myosin contractile ring that surrounds the actin cap (4). This actin cap also contains the polarity protein PAR-3 (5), with polar body extrusion in mammalian oocytes constituting an example of symmetry breaking and cell polarity (6). In both mouse and *Xenopus* oocytes, the small GTPase Cdc42 and the Arp2/3 complex are required for the assembly of branched microfilaments that constitute the actin cap and appear to push out the overlying membrane and the cortex-tethered pole during extrusion (7,8). Concurrently, the surrounding RhoA-dependent contractile ring constricts to pinch off the polar body. In addition to being required for spindle migration and polar body extrusion, actin filaments also are present throughout the mouse oocyte meiotic spindle, and their depletion results in smaller spindles and defects in chromosome separation (3).

The requirements for microfilaments and microtubules during *C. elegans* oocyte meiosis I cell division, and the dynamics of spindle movement and polar body extrusion, appear largely distinct from those reported for vertebrate oocytes. In *C. elegans*, the oocyte pronucleus moves toward the cortex during oocyte maturation, and a bipolar spindle assembles in proximity and parallel to the cortex, rather than assembling centrally as in mouse oocytes (1). Although spindle assembly begins near the cortex in *C. elegans* oocytes, the spindle nevertheless does approach the cortex more closely as assembly proceeds, with the bipolar spindle rotating during anaphase to become orthogonal to the overlying cortex, relatively late in meiosis I compared to vertebrate oocytes (9,10).

The molecular mechanisms that mediate spindle positioning in *C. elegans* also appear distinct from those operating in mammalian oocytes. In contrast to mammals, translocation and rotation of the *C. elegans* oocyte meiotic spindle do not require actin, but do require a kinesin and dynein, which presumably act on sparse but detectable astral microtubules and the cortex to properly position and orient the spindle as it shortens (9,10). Moreover, while actomyosin is cortically enriched in *C. elegans* and vertebrate oocytes, a cytoplasmic network of oocyte microfilaments, to our knowledge, has not been reported in *C. elegans*. Moreover, the entire cortical actomyosin network in *C. elegans* oocytes is highly dynamic during polar body extrusion (Movie 7), and while the contractile ring constitutes a polarization of this network, there is no evidence for overall polarization of the oocyte during meiotic cell division. Finally, it is not clear if cortical actomyosin contractility during vertebrate polar body extrusion is restricted to the contractile ring or acts more extensively throughout the cortex. In sum, among other differences, spindle positioning in *C. elegans* oocytes appears to be largely microtubule-dependent and is microfilament-independent, while in vertebrate oocytes spindle positioning is less microtubule-dependent and very much microfilament-dependent.

**S1 References**

1. Severson AF, von Dassow G, Bowerman B. Oocyte Meiotic Spindle Assembly and Function. In: Current Topics in Developmental Biology [Internet]. Elsevier; 2016 [cited 2023 Jan 4]. p. 65–98. Available from: https://linkinghub.elsevier.com/retrieve/pii/S0070215315001970

2. Uraji J, Scheffler K, Schuh M. Functions of actin in mouse oocytes at a glance. Journal of Cell Science. 2018 Nov 15;131(22):jcs218099.

3. Harasimov K, Uraji J, Mönnich EU, Holubcová Z, Elder K, Blayney M, et al. Actin-driven chromosome clustering facilitates fast and complete chromosome capture in mammalian oocytes. Nat Cell Biol. 2023 Mar;25(3):439–52.

4. Deng M, Suraneni P, Schultz RM, Li R. The Ran GTPase Mediates Chromatin Signaling to Control Cortical Polarity during Polar Body Extrusion in Mouse Oocytes. Developmental Cell. 2007 Feb;12(2):301–8.

5. Duncan FE, Moss SB, Schultz RM, Williams CJ. PAR-3 defines a central subdomain of the cortical actin cap in mouse eggs. Developmental Biology. 2005 Apr;280(1):38–47.

6. Li R, Bowerman B. Symmetry Breaking in Biology. Cold Spring Harbor Perspectives in Biology. 2010 Mar 1;2(3):a003475–a003475.

7. Dehapiot B, Carrière V, Carroll J, Halet G. Polarized Cdc42 activation promotes polar body protrusion and asymmetric division in mouse oocytes. Developmental Biology. 2013 May;377(1):202–12.

8. Zhang X, Ma C, Miller AL, Katbi HA, Bement WM, Liu XJ. Polar Body Emission Requires a RhoA Contractile Ring and Cdc42-Mediated Membrane Protrusion. Developmental Cell. 2008 Sep;15(3):386–400.

9. Fabritius AS, Ellefson ML, McNally FJ. Nuclear and spindle positioning during oocyte meiosis. Current Opinion in Cell Biology. 2011 Feb;23(1):78–84.

10. Ellefson, Marina L., McNally, Francis J. Kinesin-1 and Cytoplasmic Dynein Act Sequentially to Move the Meiotic Spindle to the OOcyte Cortex in Caenorhabditis elegans. Mol Biol Cell. 2009;20:2722–30.
