# Supplementary material for: Microtubules oppose cortical actomyosin-driven membrane ingression during C. elegans meiosis I polar body extrusion
Source: PLoS Genet. 2023 Oct 2;19(10):e1010984. doi: 10.1371/journal.pgen.1010984 (PMC10569601; doi:10.1371/journal.pgen.1010984)
Supplement: Attachment — Submitted filename: Reviewer Response.docx [file pgen.1010984.s030.docx]

Response to Reviewer Comments:

We thank the reviewers for their careful reading of our manuscript, and for their extensive, insightful, and very helpful comments and suggestions. In response, we have extensively revised our manuscript, with a substantial amount of new data now included in one new Figure and three new Supplemental Figures, and with additions to several of the original figures. We have substantially revised the title, abstract, introduction, results, section subheadings, and figure legends, to describe our results more carefully and objectively, and we include more extensive consideration of alternative interpretations and conclusions. We are grateful to the reviewers for their patience, and for their extremely helpful and insightful comments. We believe the changes we have made in response have greatly improved our revised manuscript.

Our responses below to each reviewer’s comments are highlighted in blue font, with relevant text excerpts from the manuscript also italicized.

Reviewer #1:

Polar body extrusion during female meiosis is required to produce zygotes with a correct chromosome number in nearly all animals and is thus a critically important process that requires coordination between spindle microtubules and cortical actomyosin. In this manuscript, the authors show that microtubules near the cortex far from the spindle inhibit myosin-dependent invaginations of the cortex far from the spindle. The authors speculate that their findings indicate something about how polar body extrusion at the spindle works, but this connection is not completely clear. The quality of the time-lapse imaging data, image analysis, and genetic manipulation is very high. With 3 experiments and several writing adjustments, this manuscript would be very appropriate for publication in PLoS Genetics.

Required experiments:
1.) In the introduction and results, CLS-2 is described as localizing in cortical patches that are equated with linear elements. Linear elements are ROD-1-dependent and neither polar body defects nor ectopic plasma membrane ingressions have been reported after ROD-1 depletion. The authors should test whether the cortical patches reported in Fig. 2 are dependent on ROD-1. If they are dependent on ROD-1, the authors should report on membrane invaginations in rod-1-depletions. If they are independent of rod-1, this should be clarified in the text and they should not be called linear elements.

We now include these suggested ROD-1 knockdown experiments (new SFig 7): KNL-1, BUB-1 and CLS-2 all depend on ROD-1 for localization to linear elements, based on ROD-1 RNAi depletion experiments. ROD-1 depletion also results in excess membrane ingression but polar body extrusion is usually successful. These data are now reported in two new supplemental figures (S2 and S7), and in a revised bar graph summarizing polar body extrusion defects (now S4B). We now refer to the structures as linear elements throughout the early text, but we also refer to them as sub-cortical patches after documenting their co-localizations and dependencies for patch localization, to distinguish them from the spindle-associated linear elements (see below).

The authors should also show or cite data showing a lack of colocalization of patches with myosin, actin, and a GTP-rho biosensor (because these all localize in cortical patches).

We point out in our revised “Limits of our analysis” section in the Discussion that a more extensive analysis of the spatial and temporal relationships of these different components is needed, and we are indeed pursuing such studies that we plan to publish in a future manuscript. However, we view these experiments, which also include higher resolution near TIRF microscopy, as beyond the scope of our current manuscript.

Because patches are not present during meiosis II, the authors should explain how they could be required for polar body extrusion.

In our original submission, we obliquely noted the absence of patches during meiosis II. This is now more explicitly addressed with additional Discussion text as follows:

*“Another reason to question whether the regulation of oocyte membrane ingression is important for polar body extrusion is that the KNL-1/BUB-1/CLS-2 sub-cortical patches are present only during meiosis I and are undetectable throughout all of meiosis II. While perhaps unlikely, undetectable levels of sub-cortical patches might suffice for such regulation during meiosis II. Further investigation to compare cortical actomyosin and microtubule distributions during meiosis I and II, and to assess the influence of eggshell synthesis and rigidity as meiosis proceeds, might reveal differences that preclude a need for microtubule regulation by sub-cortical patches during meiosis II.”*

2.) The data on cortical microtubule patches (CMPs) presented in Figure 5 needs some improvement in presentation because these patches are completely not obvious from the images shown. Do these cortical microtubule patches co-localize with CLS-2/BUB-1/KNL-1 cortical patches? If the same image analysis is applied to subcortical regions, are there cortical patches in the cytoplasm (not at the cortex)? What do these authors hypothesize these cortical microtubule patches represent?

We apologize for not adequately explaining the nature of what we called CMPs in our original submission. We now refer to these as sub-Cortical Microtubule Foci (sCMFs) to distinguish them from the sub-cortical patches of KNL-1/BUB-1/CLS-2. As we now state in our revised text, we view these as indicators of microtubule organization, with the brighter foci presumably representing places where microtubules cross over each other or otherwise overlap. We also now include new data (SFig4E) showing that the relative standard deviation of cortically associated microtubule signal intensity across the oocyte is decreased in *cls-2* mutants compared to control oocytes, consistent with a more even distribution of microtubules in the mutant oocytes. We further provide maximum intensity projections (MIPs) of both the surface-most focal planes and of central focal planes, to demonstrate more clearly how these microtubules are present within a layer the appears to underlie the actomyosin cortex, although they may also overlap with the cortex (we now also do this for the KNL-1/BUB-1/CLS-2 sub-cortical patches in Figure 2B). We also include surface-most and central MIPs from oocytes expressing a GFP fusion to the non-muscle myosin II NMY-2 for a direct comparison to cortical actomyosin. These new data on peripheral enrichment of microtubules are included in a new Figure 5, along with line scans that highlight the peripheral enrichment. We also have revised all other figures showing microtubule signal to include both surface-most and centrally located MIPs, and accompanying line scans. We now refer to these microtubules as “cortically associated” or as “sub-cortical” microtubules, and we avoid referring to them as a part of the actomyosin cortex. We have modified the text throughout the revised manuscript to more carefully describe our analysis of sub-cortical microtubules, and more carefully describe sCMFs as indicators of microtubule organization. The following extensively revised Results excerpt addresses these issues and concerns about our description and analysis of the oocyte microtubule cytoskeleton.

*“We previously hypothesized that CLS-2 stabilizes microtubules to promote cortical stiffness and thereby limit actomyosin-driven membrane ingression during meiosis I polar body extrusion (11) (Fig 1). We also have previously shown that microtubules distributed throughout the oocyte, outside of the meiotic spindle, are enriched near the oocyte surface (25), although we did not compare the distribution of these microtubules to the distribution of actomyosin to better assess their location relative to the oocyte cortex. Furthermore, microtubule levels near the cortex throughout the oocyte are much lower than those associated with the meiotic spindle, and we were unable in our previous study to detect any significant change in cortically associated microtubule levels in cls-2 mutant oocytes, using relatively low resolution live imaging data from in utero oocytes within immobilized whole mount worms (11).*

*To better assess whether KNL-1, BUB-1 and CLS-2 influence cortically associated microtubules during oocyte meiotic cell division, and to more closely examine the location of these microtubules relative to the oocyte cortex, we have now used higher resolution live imaging of ex utero oocytes that express GFP fused to either a β-tubulin (GFP::TBB-2) to mark microtubules, or non-muscle myosin II NMY-2 (GFP::NMY-2) to mark cortical actomyosin, and mCherry::H2B to mark chromosomes. In control oocytes, we observed a network of microtubule foci distributed throughout the surface-most focal planes, whereas within the central focal planes we observed a modest enrichment of microtubule signal near the oocyte surface that extended a few microns into the cytoplasm throughout the oocyte (Fig 5, Movie 4). By contrast, we found that all GFP::NMY-2 foci were closely apposed to the oocyte surface (Fig 5), as expected for cortical actomyosin (1,4). How intimately these cortically associated microtubules intermingle with cortical actomyosin, whether and how cortical actomyosin and these cortically associated microtubules are attached to the membrane or to each other, and the extent to which microtubules are present throughout the entire volume of the oocyte, requires further investigation (see Discussion). Nevertheless, we conclude that oocyte microtubules are enriched in a layer that appears to overlap with but largely underlies the cell cortex throughout the oocyte during meiosis I polar body extrusion. Hereafter, we refer to the cortically associated microtubules as sub-cortical.*

*To determine whether the loss of KNL-1, BUB-1 or CLS-2 might influence the sub-cortical microtubules, we used RNAi to knock down each patch component in ex utero oocytes expressing GFP::TBB-2 and mCherry::H2B. In brief, for each knockdown we observed a subtle but apparent reduction in the brightness of the sub-cortical microtubule foci scattered throughout the surface-most focal planes (Fig 6A-D, Movie 4). Furthermore, enrichment of the microtubule signal near the oocyte surface in central focal planes was lost (Fig 6E).*

*We previously reported that we could not detect a significant difference in the levels of cortically associated microtubules in cls-2 null mutant oocytes imaged in utero, based on a comparison of the integrated pixel intensities from mutant and control oocytes (11). Despite the subtly altered distribution of sub-cortical microtubules in our imaging data from ex utero oocytes reported here (Fig 6), we nevertheless have not detected a significant difference in sub-cortical microtubule levels in mutant versus control oocytes. However, based on our perception of brighter small foci of sub-cortical microtubule signal in control oocytes, we assessed the relative standard deviation of cortically associated microtubule signal intensity within the surface-most focal planes across the entire oocyte, in mutant and control oocytes at the beginning of anaphase B. We found a significant decrease in the relative standard deviation in cls-2 mutant oocytes, consistent with less variance in signal intensity across the oocyte during polar body extrusion (SFig 4E).* *We conclude that loss of CLS-2 likely leads to an alteration in microtubule organization, rather than an overall decrease in microtubule levels.*

*To further assess the organization of sub-cortical microtubules in control and mutant oocytes, we used Imaris software to define and count sub-Cortical Microtubule Foci, or sCMFs, during polar body extrusion (see Materials and Methods). In brief, sCMFs were defined based on the local variance of pixel intensity above threshold variance values, counted throughout the merged surface-most focal planes at each time point, and divided into weak, medium and strong sCMFs based on relative pixel intensity (Fig S6, Movie 5). We suspect that these sCMFs represent places in which multiple microtubules in some way overlap or cross over each other within the sub-cortical region and thus provide an assessment of microtubule organization. We observed significant decreases in the number of medium sCMFs, and in the total number of all sCMFs, after KNL-1 and BUB-1 RNAi knockdowns and in cls-2(or1948) oocytes, compared to control oocytes (Fig 7).”*

3.) Excessively deep membrane invaginations in knl-1(RNAi) or bub-1(RNAi) were indistinguishable from those in cls-2(RNAi) but polar body extrusion occurred in 80% of embryos. It is suggested that this is due to partial knockdown by RNAi, however, the simple interpretation is that limiting membrane invaginations through BUB1/KNL-1 is not required for polar body extrusion. If the authors wish to make an argument based on partial depletion, they should show the extent of depletion. Either the authors need to achieve a higher degree of knockdown, or the overall significance of regulating invaginations over the entire embryo requires more careful explanation.

We thank the reviewer for pointing out this significant flaw in our logic. We had in fact considered this lack of correlation as a concern but did not acknowledge it our original manuscript (a clear case of bias intruding into how we wrote our manuscript). We now include new data on polar body extrusion after degron tagging both the *knl-1* and *bub-1* endogenous loci, for auxin-mediated degradation of the encoded proteins, which often results in equally or more severe depletion compared to RNAi. While we observed a much more highly penetrant polar body extrusion defect for *bub-1(AID)* oocytes, the *knl-1(AID)* oocytes still extruded a polar body in 90% of the depleted oocytes. We also now provide quantification of the extent of depletion for our RNAi knockdowns for KNL-1, BUB-1 and CLS-2, and also for ROD-1 RNAi knockdown (in a new supplemental figure S2 and also in a new panel, S5D). We also now include a much more explicit account of the lack of correlation between excess membrane ingression and polar body extrusion in a new section our revised Discussion as follows.

***“The relationship between oocyte membrane ingression and polar body extrusion***

*While our results clearly indicate that oocyte microtubules influence membrane ingression throughout the oocyte during polar body extrusion, the functional importance of this regulation is much less clear. Because the contractile ring during C. elegans oocyte cytokinesis forms within a much larger and actively contractile cortical actomyosin network that extends throughout the oocyte (Fig 1), we speculate that a proper balance of cortical actomyosin tension and sub-cortical microtubule stiffness modulates cortical actomyosin dynamics throughout the network to promote ring assembly and ingression. Indeed, in klp-7, zyg-9 and cls-2 mutant oocytes, a contractile ring often begins to assemble but then becomes distorted and usually collapses into a single prominent furrow that ultimately regresses with failed polar body extrusion. It is tempting to infer that altered sub-cortical stiffness in mutant oocytes puts a strain on the cortical actomyosin network that disrupts contractile ring assembly and ingression.*

*If our hypothesis that strain within the cortical actomyosin network disrupts contractile ring dynamics were correct, we would expect mutant oocytes with similarly excessive membrane ingression to fail in polar body extrusion with similar penetrance. However, while we observed quantitatively similar membrane ingression defects in rod-1, knl-1, bub-1 and cls-2 mutant oocytes, they differed substantially with respect to the penetrance of their polar body extrusion defects. Over 1/3 of null cls-2(or1948) oocytes succeeded in polar body extrusion as we scored it, while only 10% of bub-1(AID) oocytes succeeded, and 90% or rod-1(RNAi) oocytes and 75% of knl-1(AID) oocytes succeeded in polar body extrusion. More complete depletion of these proteins, and a detailed examination of oocyte meiotic contractile ring dynamics, might reveal shared defects. Nevertheless, the similar membrane ingression defects, in mutants with very different rates of failure in polar body extrusion, argues against excess strain throughout the cortical actomyosin network interfering with polar body extrusion.*

*Regardless of the relationship between oocyte membrane ingression and polar body extrusion, our results show that highly regulated microtubules are enriched in proximity to the plasma membrane, where they appear to modulate the extent of membrane ingression throughout the oocyte during meiosis I polar body extrusion.”*

Documentation of the extent of knockdown of csnk-1 is also essential for the conclusion made in the discussion based on a lack of phenotype for csnk-1(RNAi).

We were unable to obtain a viable strain of a GFP fusion to *csnk-1* from the *C. elegans* stock center to assess the degree of CSNK-1 knockdown after our RNAi treatments. Due to this lack of quantification, and to the tangential nature of these results, we have removed all of our data on CSNK-1 and now only briefly refer to the possible role of CSNK-1 in our revised Discussion.

Major improvements to writing:

In the introduction, the authors should bring up the two possible mechanisms by which microtubules might inhibit actomyosin-dependent invagination of the plasma membrane, direct coupling by mechanical attachment of microtubules to the plasma membrane or inhibition of actomyosin by microtubules. It is well known that astral microtubules emanating from centrosomes regulate cortical actomyosin but this is not mentioned until Fig. 9 of the results and is inappropriately discounted in the discussion. This more unbiased writing is especially important since no data directly measuring cortical stiffness is presented and the authors never clearly state how microtubules would generate stiffness. The possible role of osmotic pressure in stiffness should also be brought up in a more unbiased manner.

We thank the reviewer for suggesting a less biased presentation of our results. We have modified the text throughout to remove our previous bias. We no longer refer to oocyte microtubules as being part of the cortex, or cortical. We now include a much more balanced consideration of how microtubules and actomyosin might be interacting during polar body extrusion. We also now provide a more comprehensive consideration of the roles of cross-linking to the membrane, and possible cytoskeletal cross-linking, which we did not address in our original submission. To address these concerns, we have added next text to the Results, and a new section to our Discussion entitled “Anchoring of cortical actomyosin and sub-cortical microtubules in *C. elegans*  oocytes,” as follows.

New Results text:

*“How intimately these cortically associated microtubules intermingle with cortical actomyosin, whether and how cortical actomyosin and these cortically associated microtubules are attached to the membrane or to each other, and the extent to which microtubules are present throughout the entire volume of the oocyte, requires further investigation (see Discussion). Nevertheless, we conclude that oocyte microtubules are enriched in a layer that appears to overlap with but largely underlies the cell cortex throughout the oocyte during meiosis I polar body extrusion. Hereafter, we refer to the cortically associated microtubules as sub-cortical”*

New Discussion section text:

*“****Anchoring of cortical actomyosin and of sub-cortical microtubules in C. elegans oocytes***

*A key property of cortical actomyosin is its attachment to the overlying plasma membrane, which links tension and elasticity in the cell cortex to the membrane, and to cell shape and morphogenesis (1,4)****.*** *One important mechanism for attachment to the membrane occurs through Ezrin/Radixin/Moesin (ERM) family members that bind both to microfilaments and to a variety of membrane proteins (40,41). The increased cross-linking of cortical microfilaments to the membrane by ERM family members, during insect and animal cell division, reduces elasticity to stiffen the cortex and promote mitotic rounding (6–8). Myosin 1 and filamin family members also have been implicated in attaching cortical microfilaments to the overlying membrane in some cell types (41–44).*

*The conservation of cortex/membrane attachment by ERM, Myosin 1, and filamin family members in different animal phyla and cell types has not been extensively explored. While we have shown that cortical actomyosin is coupled to the membrane in early embryonic blastomeres in C. elegans (45), the molecules that mediate attachment are not known. The C. elegans genome encodes only a single ERM family member, MOE-1, with limited expression in several larval cell types and limited post-embryonic requirements during larval intestinal lumen and vulval development (46). Similarly, the two Myosin 1 family members in C. elegans, HUM-1 and -5, and the two C. elegans filamin family members, FLN-1 and -2, are expressed outside of the germline in a limited number of cell types and have limited post-embryonic requirements (47–49). Further investigation is needed to identify C. elegans factors that attach cortical microfilaments to the cell membrane, not only in oocytes but throughout development.*

*How oocyte microtubules and the KNL-1/BUB-1/CLS-2 patches are restricted to a sub-cortical layer throughout the oocyte, and whether these microtubules and sub-cortical patches extend into and intermingle with or are molecularly linked to cortical actomyosin or to the cell membrane, also is not clear. One appealing candidate for linking sub-cortical microtubules to cortical actomyosin is dynein, which has been implicated in coordinating interactions between the microtubule and microfilament cytoskeletons in other contexts, including the early C. elegans embryo (50,51). Whether C. elegans dynein influences the distribution of oocyte sub-cortical microtubules or the KNL-1/BUB-1/CLS-2 sub-cortical patches, or affects membrane ingression during polar body extrusion, requires further investigation.”*

Throughout the manuscript, the use of the words “stiffness” and “elasticity” and the apparent assumption of an inverse relationship between them is confusing and somewhat over-stated. Neither physical parameter is measured experimentally here (for example by needle deflection experiments that have been published for mouse oocytes). Stiffness has units of N/m. The inverse of stiffness, compliance, has units of m/N. Elasticity has units of N/m2. Several sentences in the manuscript do not make sense. This reviewer understands how stiffness could resist deformation (invagination) by myosin contractility. This reviewer does not understand the assumed relationship with elasticity. It is up to the authors to explain this relationship.

We apologize for our naïve account of elasticity and stiffness. We no longer refer to elasticity with respect to the oocyte sub-cortical microtubules but rather refer to them as possibly providing a sub-cortical stiffness that resists cortical actomyosin-driven membrane ingression.

This manuscript addresses the function of “cortical microtubules” implying that there is a subset of microtubules that are in some way attached to the cortex. Because microtubules fill the entire volume of the meiotic embryo and there is no evidence of a mechanical attachment between microtubules and the plasma membrane and typical minus-end binding proteins do not localize at the cortex, the authors need to more carefully describe what is known about the polarity of cortical or cytoplasmic microtubules. Cortical microtubules have been characterized in Drosophila oocytes and epithelial cells of C. elegans and vertebrates. In all of these cases, g-tubulin is concentrated at the cortex implying attachment of microtubule minus ends to the cortex. G-tubulin does not localize to the cortex of C. elegans meiotic embryos making the polarity and mechanical attachment unclear. Minus ends at the cortex have been suggested by the phenotype of kinesin-1 mutants by McNally 2010 Dev Bio 339:126 and Kimura 2017 Nat Cell Bio 19:399.

As noted earlier, we no longer refer to cortical microtubules but to cortically associated or sub-cortical microtubules, based on our inclusion of new data indicating an enrichment of these microtubules that for the most part underlies the actomyosin cortex. We do not have data as to the polarity of these microtubules or their nucleation, and we make it clear that any cross-linking of these sub-cortical microtubules to the overlying membrane or the actomyosin cortex requires further investigation, as noted above.

Minor improvements to writing:

The subheading “KNL-1, BUB-1 and CLS-2 stabilize cortical microtubules during meiosis I polar body extrusion” should be changed to “KNL-1, BUB-1 and CLS-2 increase the number of cortical microtubule patches during meiosis I polar
body extrusion” since no measure of microtubule stability is shown.

We thank the reviewer for pointing out our overstatements as to microtubule stability (or indeed levels); we no longer include this subheading, and the text in this section has been mostly deleted or is included under other, more appropriate subheadings. As detailed in our response to required experiment #2, we have revised our text to indicate that we view our measurement of sub-cortical microtubule foci (sCMFs) as an indicator of microtubule organization, not of microtubule stability or overall levels. We have made changes throughout the manuscript to avoid referring to any requirement for microtubule stability or levels, but rather to requirements for the organization of sub-cortical microtubules.

No mutant that specifically eliminates CLS-2/BUB-1/KNL-1 patches is described making it difficult to assess the significance of these patches.

In our original submission we noted that we could not rule out other pools of these proteins mediating the regulation of sub-cortical microtubules. We now include this point in both the Results and the Discussion of our revised manuscript, as follows.

Results text: *“While we cannot exclude other pools of these proteins being responsible (see Discussion), the sub-cortical patches are well positioned to regulate microtubules that limit membrane ingression throughout the oocyte during meiosis I polar body extrusion.”*

Discussion text: *“While we hypothesize that the KNL-1/BUB-1/CLS-2 patches regulate sub-cortical microtubule dynamics, we cannot rule out the alternative possibility that spindle- and chromosome-associated, cytoplasmic, or other pools of these patch proteins influence sub-cortical microtubule dynamics. Optogenetic experiments designed to recruit sub-cortical patch components, or a heterologous microtubule destabilizing activity called Optokatanin (61), to limited areas of the oocyte cortex may provide more conclusive evidence that localized modification of microtubule dynamics can influence local membrane ingression independently of changes in cortical actomyosin dynamics.”*

Fig. 3B needs quantification and more explanation. How many embryos displayed the phenotype shown? Measurements of fluorescence intensity showing the variability would be more rigorous than just stating a black and white dependence. This analysis should also show the extent of depletion of the protein targeted by RNAi. It is not possible to conclude that KNL-1 localization is independent of CLS-2 unless complete loss of CLS-2 is demonstrated. The legend states that the images shown were acquired at “roughly metaphase”. A brief explanation of how metaphase was scored (especially in the knl-1 depletion) would make the result more reproducible by others.

We were remiss in not quantifying these results and now include data showing the effective of our RNAi knockdowns in depleting the targeted proteins, and in reducing the sub-cortical patch GFP signal in our knockdowns that assess dependency for patch localization (new SFig 2). As the reviewer accurately pointed out, we in fact do see a substantial degree of mutual dependence for patch localization, rather than the simpler linear relationship reported at kinetochores. We thank the reviewer for this improvement to our manuscript and have modified the text to describe these results more accurately as follows.

In revised Figure 3 legend we more carefully describe how we defined metaphase:

*“All images are from ex utero oocytes during metaphase, shortly before anaphase chromosome movement, when the patches are numerous and bright.”*

Modified Results text:

*“At kinetochores, CLS-2 requires both KNL-1 and BUB-1 for its localization, while BUB-1 requires only KNL-1, and KNL-1 does not depend on either BUB-1 or CLS-2 (16).To assess their dependencies at sub-cortical patches, we used RNA interference (RNAi) to knock down each component in oocytes expressing GFP fusions to one of the three proteins, to assess both the effectiveness of our RNAi knockdowns and the requirements for sub-cortical patch localization (see Materials and Methods). Our RNAi knockdowns did not completely reduce the levels of the proteins targeted for depletion, with signal still detectable in association with chromosomes (Fig 3B). However, when we quantified the signal detected in sub-cortical patches throughout the five surface-most focal planes during five consecutive time points prior to the start of anaphase B, we found that each RNAi knockdown nearly or completely eliminated patch signal intensity for the targeted protein (SFig 2). We found that CLS-2 was undetectable in cortical patches after knocking down either KNL-1 or BUB-1 (Figs 3B, S2), consistent with their relationships at kinetochores. For BUB-1, about 90% of the BUB-1::GFP patch signal was lost after KNL-1 knockdown, much as has been observed at kinetochores, with the residual BUB-1 perhaps being due to incomplete knockdown. However, we also saw a roughly 50% reduction in BUB-1 patch signal after CLS-2 knockdown (Figs 3B, S2), whereas as BUB-1 has been reported to localize to kinetochores independently of CLS-2 (16). Finally, while KNL-1 was reported to localize to kinetochores independently of both BUB-1 and CLS-2 (16), we found that KNL-1 patch levels were reduced by nearly 50% after BUB-1 knockdown, and we saw some reduction in KNL-1 patch signal after CLS-2 knockdown (Figs 3B, S2). To summarize, during C. elegans oocyte meiosis I cell division, the kinetochore proteins KNL-1, BUB-1 and CLS-2 co-localize to dynamic sub-cortical patches that appear upon nuclear envelope breakdown and persist until the beginning of anaphase B during meiosis I, with greater mutual dependence for patch localization than has been reported for kinetochores.”*

Adding page and line numbers to the manuscript would make life easier for a reviewer.

We now include page and line numbers throughout.

In most of the manuscript, references are called out with a number while in some locations references are listed by author name and year.

Our apologies. All references have been formatted properly in the revised manuscript.

Some of the writing needs to be condensed (eg. the legends for Fig. 1 and 4, and the discussion).

We have shortened both figure legends, and the other figure legends. The Discussion has been shortened by 29% (from 3907 to 2767 words), and we have made the text more concise throughout.

The authors should at least comment on whether the plasma membrane marker (PH domain of rat phospholipase C zeta which binds specifically to PIP2) could be influencing their results. At a minimum, a description of the properties of this marker should be included.

We now include a description of this marker and address potential concerns as follows.

*“Because chromosome separation fails in cls-2 mutant oocytes (11,14,16), we defined the beginning of anaphase B as the timepoint when the oocyte chromosome mass was most condensed (31), for all control and mutant oocytes. The GFP::PH fusion encodes a rat phospholipase C PH domain that is known to bind PIP2 membrane lipids (32). While this domain might interfere with cortical dynamics, the membrane ingressions observed in control and mutant oocytes resemble those observed in oocytes that lack the GFP::PH fusion, using Nomarski optics.”*

How is the onset of anaphase B determined in strains expressing mCherry::H2b and GFP::PH when distinguishing between anaphase A and anaphase B requires imaging the spindle poles? The results text states that maximum chromatin compaction was used for cls-2-depleted embryos but the legend implies this was used for all genotypes. This should be clarified.

We apologize for not making it clear that we used chromosome compaction to score the onset of anaphase B in all genotypes, and this has now been clarified as shown in the revised text quoted in response to the previous comment. We also repeat this point in subsequent text and in the figure legends.

“Taxol treatment substantially elevated cortical microtubule levels during meiosis I (Fig 6A, Movie 3)” should show some quantitation rather than just a single image.
Polar body extrusion appears normal without membrane invaginations in taxol-treated oocytes, again raising the question of significance of the invaginations around the entire embryo.

We were remiss in not quantifying the effects of not only taxol but also nocodazole on microtubule levels in our original manuscript and have now done so in a new panel, SFigure 7D. As discussed above, we also now include a more objective discussion about the lack of correlation between increased oocyte membrane ingression and polar body extrusion. Note that while our nocodazole treatments did significantly reduce raw integrated pixel intensity for microtubules throughout the oocytes (at ¾ of the time through anaphase B), we saw more variable effects with taxol that were not statistically significant. We thank the reviewer for pointing out this omission and we have revised two Results sections describing these new data as follows.

*“We next asked if chemically stabilizing oocyte microtubules with taxol would reduce the extent of membrane ingression during polar body extrusion. Taxol treatment variably elevated sub-cortical microtubule levels during meiosis I to an extent that was not statistically significant in our small sample size of five oocytes (Fig 8A, S7C,D, Movie 3). Nevertheless, taxol treatment did significantly reduce the extent of membrane ingression during anaphase B (Fig 8A,B, Movie 3), although polar bodies were still extruded in about 2/3 of taxol treated oocytes (Figs 8B, S4B). The increased and decreased membrane ingressions observed after nocodazole and taxol treatment, respectively, further support our hypothesis that sub-cortical microtubules limit membrane ingression during meiosis I polar body extrusion…*

*… Finally, polar body extrusion always failed after nocodazole treatment to destabilize microtubules, but failed in only about 1/3 of oocytes exposed to taxol treatment to stabilize them (SFig 4B), although our taxol treatments may not have elevated microtubules sufficiently to interfere with polar body extrusion in some oocytes (SFig 7D). Though variable, these results are consistent with sub-cortical microtubule organization and levels being important for polar body extrusion in C. elegans oocytes.”*

Fig. 7G right 2 panels have a comparison of 3 data sets but only one significance. This needs to be clarified especially for the far right. The conclusion in the text is that cls-2; klp-7 did not have a significant effect but the figure shows p<.01. This needs to be clarified.

We apologize for mis-stating these results. As the reviewer accurately notes, there is a significant reduction in *cls-2 klp-7* double mutants compared to *klp-7* mutants, while there is not a significant difference for the *zyg-9* double and single mutant oocytes. We now describe this accurately as follows.

*“While KLP-7 and ZYG-9 knockdowns both led to increased levels of sub-cortical microtubules, the microtubule properties in each mutant were distinct. While the number of sCMFs in zyg-9 mutants did not show a statistically significant dependence on CLS-2, the sCMFs were partially but significantly reduced in klp-7 cls-2 double mutants relative to klp-7 single mutants (Fig 9H). Nevertheless, sCMFs in both double mutants were still significantly elevated compared to control and cls-2 mutants (Fig 9H).”*

The lack of a phenotype after csnk-1(RNAi) used as evidence that hydrostatic pressure is not involved in polar body extrusion is beyond mystifying.

We regret having implied that hydrostatic pressure is not involved in polar body extrusion when we referred to other possible roles for cortical actomyosin in our original discussion. In fact, we only meant to question previously published work from the McNally lab showing that loss of CSNK-1 increases cortical actomyosin contractility and hydrostatic pressure. We did not observe the expected increase in membrane ingression in our CSNK-1 knockdown experiments, in contrast to those results reported (but not quantified) by the McNally lab. However, as mentioned earlier, we have removed all of our analysis of CSNK-1 from this manuscript as it is peripheral to our conclusions. Also, we would want to more extensively examine *csnk-1* mutant oocytes, including verifying the extent of our knockdowns, before reaching conclusions contrary to what has already been published. We no longer make any reference to the role of hydrostatic pressure during polar body extrusion, and we now refer to CSNK-1 only once, in the Discussion, as a possible target of KNL-1/BUB-1/CLS-2 regulation.

Reviewer #2:

Quiogue et al address the role of non-chromosome associated outer kinetochore protein assemblies on membrane ingression during C. elegans meiosis I. This is a very interesting subject because since their identification decades ago, these patches, rods, or linear elements, as they have been referred to, their function has not been addressed. The authors of this manuscript focus on a subset of these proteins, the outer kinetochore scaffold, KNL-1, the kinase BUB-1, and the microtubule stabilising protein CLS-2.

The authors show that KNL-1, BUB-1, and CLS-2 are required to limit membrane ingression during meiosis I. Ultimately, the authors suggest that CLS-2 as part of these non-chromosomal outer kinetochore patches, stabilises microtubules and hence stiffen the oocyte cortex, limiting membrane ingression.

Even though it is not clear how these patches could regulate microtubule stability (which do not co-localise with cortical microtubules), the observations are interesting and are likely to contribute to further investigate and understand the role of these elusive protein assemblies. The experiments are well performed and thoroughly analysed. Furthermore, there is ample explanation on the quantitative analysis. I have some questions/comments that the authors might want to look into, but I am overall in favour of publication.

- The key big question is how the authors envision that these patches regulate cortical MTs and what would be the ‘anchor’ for these patches, since presumably CENP-A and CENP-C are not present. Is MEL-28 required for KNL-1?

We agree this is a key question, which we now address much more extensively. As summarized in responses to the Reviewer #1, we have now documented in more detail how both the KNL-1/BUB-1/CLS-2 patches and the non-spindle microtubules are distributed throughout the oocyte in proximity to the cortex but mostly appear to underlie the cortex. We also have examined their localization and dynamics after nocodazole treatment and to our surprise found that they appeared to become longer and more numerous, although we have not quantified this difference (data shown in new Movie 6). As also pointed out in response to Review #1, we now provide a much more extensive account of how little is known about the crosslinking of microfilaments to the membrane in *C. elegans,* and now discuss how cortical actomyosin and sub-cortical microtubules might interact with each other and with the patches. However, we view further investigation of these questions, and further investigation of the many different linear element components and their roles, as beyond the scope of this manuscript.

- The authors mention in the introduction that CLS-2 is part of a kinetochore sub-complex that includes BUB-1 and HCP-1/2. While BUB-1 can interact with HCP-1/2 and the latter with CLS-2, we don’t know whether this is a subcomplex and if so, its composition. Additionally, the co-localisation of BUB-1, HCP-1/2, and CLS-2 is highly dynamic, and it is lost as anaphase progresses. Therefore, pointing to these specific proteins as a complex might give the reader the wrong impression that these proteins interact throughout meiosis I.

We thank the reviewer for this suggestion. We indeed overstated their presence as being in various complexes and have revised our manuscript to simply describe where they localize without implying how they might or might not interact at those sites. We also now refer to them as a “group of kinetochore proteins” rather than as a “complex,” in the abstract and throughout the manuscript. The only time we refer to them as being in any complex is as an outer kinetochore sub-complex.

- Similar point as above, when the authors mention that BUB-1 and CLS-2 but not KNL-1 localise to central spindle, this is not the full picture, since the two proteins occupy different regions within the segregating chromosomes and are therefore likely not forming a complex at this stage. They might be interacting in the patches, which might show different dynamics than spindle or chromosome-associated kinetochore proteins.

We agree and have revised the text to avoid referring to them as being in complexes.

- In Figure 3B, care should be taken when assessing the dependencies for each protein localisation since this is being analysed with transgenes (in the background of untagged endogenous protein) and their levels might differ significantly from the endogenous proteins. Maybe using endogenously tagged proteins and/or immunofluorescence would be important here. Additionally, the fact that depletions are partial complicates the analysis. And lastly, it appears as if bub-1(RNAi) reduces the KNL-1 cortical signal but the authors say the opposite. Some form of quantitation would make it easier for the reader to follow.

See response to Reviewer #1 as to our new quantification data on this point.

- Still in Figure 3, do KNL-1 and BUB-1 localise normally in cls-2 mutant? This is important because the authors switch from RNAi to the mutant at this point in the paper.

We have not examined KNL-1 or BUB-1 in the *cls-2(or1948)* null mutant, although our quantification does show at least a partial dependence of BUB-1 on CLS-2, and of KNL-1 on both BUB-1 and CLS-2 after RNAi knockdowns, as also noted in response to Reviewer #1.

- In Figure 4, there seems to be significant ingression in or around the spindle area in bub-1(RNAi) and knl-1(RNAi) oocytes, but not in cls-2 mutant oocytes, where these ingressions appear outside the spindle region. Additionally, while the image shown for knl-1(RNAi) shows a great degree of ingression in the anterior region, the quantitation shows no significant different vs control. Is this not a representative image?

The patterns of ingression are indeed variable; the multiple time projections shown for each genotype in Figure S2 provide a more extensive comparison, with substantial variability in all genotypes, although we have not quantified this spatial variation. We have revised the text describing their ingression phenotypes, referring to this variability, as follows.

*“After RNAi knockdown of either KNL-1 or BUB-1, we observed abnormally deep membrane ingressions that were variably patterned and indistinguishable from those observed in cls-2 mutants (Figs 4C,D, S3, Movie 3).”*

- In Figure 6, does nocodazole treatment affect KNL-1, BUB-1, or CLS-2 localisation? Does taxol treatment rescue the defect upon bub-1(RNAi), knl-1(RNAi), or cls-2(or2948)?

We thank the reviewer for these suggestions. As noted above in response to Reviewer #1, we now include data showing that the GFP::KNL-1-labeled patches appear become longer and numerous after nocodazole treatment (to our surprise; we thought they might dissipate), as shown in new SFig 5. And taxol treatment does suppress the excess ingression in *cls-2(or1948)* oocytes, as shown in new SFig 7.

- The authors point to the RZZ complex in the discussion (without mentioning it specifically). Given the ability of the RZZ complex to oligomerise, could the RZZ be the ‘scaffold’ of these patches? Have the authors tested RZZ components localisation and/or the effect of their depletion on cortical MTs?

See response to Reviewer #1as to our new data on ROD-1 also being required for KNL-1 sub-cortical patch localization, and to limit oocyte membrane ingression.

- Check citations format. Some appear as numbers, some are cited by name, and also the text ‘(REF)’ appears.

Fixed.

**Have all data underlying the figures and results presented in the manuscript been provided?**
Large-scale datasets should be made available via a public repository as described in the *PLOS Genetics*[data availability policy](https://urldefense.com/v3/__http:/journals.plos.org/plosgenetics/s/data-availability__;!!C5qS4YX3!DbflgP2Taymvd3Zqwl0Yh8zBqTVZ_Zyv6vWMK1uzAJXXE_nub5f723OqG39LOeMkBD0W2MkuI6Cqck_GAHM$), and numerical data that underlies graphs or summary statistics should be provided in spreadsheet form as supporting information.

Reviewer #1: Yes

Reviewer #2: Yes

PLOS authors have the option to publish the peer review history of their article ([what does this mean?](https://urldefense.com/v3/__https:/journals.plos.org/plosgenetics/s/editorial-and-peer-review-process*loc-peer-review-history__;Iw!!C5qS4YX3!DbflgP2Taymvd3Zqwl0Yh8zBqTVZ_Zyv6vWMK1uzAJXXE_nub5f723OqG39LOeMkBD0W2MkuI6CqxeoX5qc$)). If published, this will include your full peer review and any attached files.


If you choose “no”, your identity will remain anonymous but your review may still be made public.


**Do you want your identity to be public for this peer review?** For information about this choice, including consent withdrawal, please see our [Privacy Policy](https://urldefense.com/v3/__https:/www.plos.org/privacy-policy__;!!C5qS4YX3!DbflgP2Taymvd3Zqwl0Yh8zBqTVZ_Zyv6vWMK1uzAJXXE_nub5f723OqG39LOeMkBD0W2MkuI6CqEwETxyQ$).

Reviewer #1: No

Reviewer #2: **Yes:** Fede Pelisch
